# Genome-Wide DNA Methylation Analysis of Performance Variation in the 5000-m Speed Race of Yili Horses

**DOI:** 10.3390/ani16020302

**Published:** 2026-01-19

**Authors:** Dehaxi Shan, Xinkui Yao, Wanlu Ren, Qiuping Huang, Yi Su, Zexu Li, Luling Li, Ran Wang, Shikun Ma, Jianwen Wang

**Affiliations:** 1 College of Animal Science, Xinjiang Agricultural University, Urumqi 830052, China; 18093792263@163.com (D.S.); yaoxinkui@xjau.edu.cn (X.Y.); 13201295117@163.com (W.R.); 18582253807@163.com (Q.H.); 13335339131@163.com (Y.S.); 13593312012@163.com (Z.L.); 18996888638@163.com (L.L.); 17590811761@163.com (R.W.); 18299152719@163.com (S.M.); 2 Xinjiang Key Laboratory of Equine Breeding and Exercise Physiology, Urumqi 830052, China

**Keywords:** Yili horse, genome-wide bisulfite sequencing, DNA methylation, differentially methylated regions, differentially methylated sites

## Abstract

Whole-genome bisulfite sequencing (WGBS) was employed in this article to map blood DNA methylation profiles at single-base resolution in Yili horses before a 5000 m speed race, with comparative analysis of epigenetic differences between the ‘elite group’ and ‘ordinary group’ across six four-year-old stallions. The overall methylation level in the elite group was generally higher than that in the ordinary groups, with a minority of regions showing hypomethylation. For instance, the promoter regions of key metabolic and neuro-related genes exhibited significant hypomethylation. The article identified over 10,000 CG differential methylation regions (DMRs), predominantly enriched in promoter and CpG island regions, anchoring 7221 differentially methylated genes (DMGs). These DMGs were significantly enriched in key biological processes including oxidative phosphorylation, protein binding, axon guidance, glutamatergic synapses, and the Hedgehog signalling pathway. Among these, six genes—ACTN3, MSTN, FOXO1, PPARGC1A, ND1, and ND2—were selected as core candidate genes closely associated with muscle strength, energy metabolism, and stress adaptation. The study confirms that the differences in athletic ability among Yili horses have a significant epigenetic basis, with DNA methylation participating in the epigenetic regulation of athletic traits by modulating the expression of genes related to energy metabolism and neuroplasticity. The constructed “promoter hypomethylated DMR panel” holds promise for translation into non-invasive blood-based epigenetic markers for early performance evaluation and targeted breeding in racehorses. This provides a theoretical basis and molecular targets for improving equine athletic phenotypes and optimising training strategies.

## Simple Summary

Employing whole-genome bisulfite sequencing (WGBS), we generated the first single-base-resolution methylome of pre-race jugular blood in Yili horses (*n* = 6) contesting a 5000 m speed trial. High—versus average—performers exhibited > 10,000 CG-biassed differentially methylated regions (DMRs) concentrated in promoter and CpG-island landscapes. Hypomethylated DMRs in elite animals significantly targeted nuclear (PPARGC1A) and mitochondrial (ND1/ND2) oxidative phosphorylation genes together with neuro-muscular plasticity loci (axon guidance, glutamatergic synapse), indicating an epigenetically primed energy–neural axis conducive to explosive ATP provision and rapid motor unit recruitment. The data validate blood-derived DNA methylation signatures as non-invasive epigenetic biomarkers for early selection and tailored training of racing stock and provide novel molecular targets for precision breeding within the Yili horse population.

## 1. Introduction

Thoroughbred and warm-blood speed disciplines are among the most capital-intensive sectors of the global equine industry; incremental gains in racetrack performance therefore hinge on precise elucidation of the physiological trade-offs between maximal exertion and long-term welfare. Recent evidence implicates epigenetic modifications—particularly DNA methylation—as the principal molecular interface through which extrinsic stressors (training load, transport, ambient temperature) are transduced into durable alterations in gene expression and metabolic efficiency [1]. Although haematological and biochemical indices have been exhaustively profiled across endurance and sprint formats, a genome-wide, nucleotide-resolution map of exercise-induced methylation dynamics, and its linkage to performance phenotypes, remains unavailable for the Chinese Yili horse.

Cytosine methylation at CpG dinucleotides is a stable yet reversible epigenetic mark that, in conjunction with post-translational histone modifications, governs chromatin architecture and transcriptional accessibility [1]. DNA methylation modifications predominantly occur at cytosine residues and can be taxonomically classified into three distinct categories based on sequence context: CpG methylation, CHG methylation, and CHH methylation. Specifically, CpG methylation denotes the modification pattern occurring within a cytosine-guanine dinucleotide motif (C–G), which constitutes the principal epigenetic mark in mammalian somatic cells of higher vertebrates, representing over 95% of total methylated cytosine residues. In contrast, CHG methylation (where C is followed by a non-guanine base H = A/C/T, then G, e.g., CAG, CCG, CTG) and CHH methylation (where C is followed by two successive non-guanine bases, CAA, CAT, CTT) are collectively designated as “non-CpG methylation.” The modification abundance of non-CpG methylation in mammalian somatic tissues is substantially lower than that of CpG methylation, typically comprising less than 1% of total methylation sites.

Tissue-and developmental-stage-specific methylation patterning is well documented in mammals, but comparable reference data are scarce for equine skeletal muscle or blood leukocytes—tissues routinely sampled in performance testing. In both human athletes and murine models, acute bouts of high-intensity interval exercise (HIIE) or chronic aerobic training demethylate promoter/intron regions of master metabolic regulators (PGC-1α, AMPKα, PPARδ) [2], thereby up-regulating oxidative phosphorylation (OXPHOS) genes and promoting slow-twitch myofibre expansion. Analogous observations have been reported in the horse: eight weeks of incremental treadmill work reduced PPARGC1A promoter methylation by 18.3% and increased mRNA abundance 2.1-fold in the gluteus medius of Standardbred horses, whereas a 90 km endurance race induced hypomethylation of the glucocorticoid receptor gene (NR3C1) in Arabian blood, correlating inversely with post-exercise cortisol [3]. Collectively, these studies suggest that DNA methylation participates in the adaptive reprogramming of equine energy metabolism and hypothalamic–pituitary–adrenal (HPA) axis recovery; however, they are constrained to candidate loci or single time-points, precluding a systems-level understanding of methylation-mediated performance regulation.

In this study, the utilisation of pre-race blood to analyse and predict post-race performance is scientifically grounded in the exploration of “epigenetic potential” rather than “acute stress response.” The core objective is not to capture dramatic physiological fluctuations on race day, but to investigate whether inherent and stable epigenetic differences exist among individuals under pre-competition steady-state conditions, which constitute the molecular basis of their athletic potential. Blood samples collected after 12 h of pre-race rest maximally exclude interference from acute race stimuli, thereby better representing the basal epigenetic characteristics of individuals in a resting state. As a relatively stable epigenetic modification, DNA methylation can chronicle the “history” of long-term training and adaptation. The “epigenetic pre-adaptation” hypothesis proposed in this study posits that elite racehorses exhibit a mosaic pattern of “global hypermethylation coupled with focal hypomethylation of key gene promoters” prior to competition, which may represent an “epigenetic memory” imprinted by long-term conditioning. This state implies that the genomes of superior individuals have been epigenetically programmed into a “primed and ready” stage—wherein non-essential genes are suppressed while key genes involved in energy metabolism and neuromuscular coordination (such as PPARGC1A and neural plasticity-related genes) are rendered more susceptible to rapid activation. Consequently, the stable pattern detected in pre-race blood serves as a molecular manifestation of their exceptional athletic potential. Although athletic performance is primarily determined by organs such as skeletal muscle, blood, as the circulatory system, can carry long-term adaptive signals from the entire body. Research indicates that systemic metabolic adjustments and stress axis regulation resulting from long-term training are reflected in the epigenetic patterns of blood leukocytes; simultaneously, metabolites and exosomes released from exercising muscle can enter the circulation, influencing the epigenetic state of blood cells and thereby leaving a “training imprint” in the blood. Existing literature supports the concordance of methylation change trends between blood and muscle at specific gene loci, validating the feasibility of using blood as a non-invasive surrogate tissue to explore performance-related epigenetic signatures. From a study design perspective, the advantage of pre-race sampling lies in its ability to distinguish causal relationships: sampling only post-race would preclude determination of whether methylation differences are the cause or consequence of superior performance. Pre-race sampling provides temporal evidence supporting the notion that “epigenetic signatures may be inherent determinants of performance variation.” Ultimately, the applied objective of this research is to develop non-invasive biomarkers for early selection and personalised training regimens, and only stable markers detectable prior to competition possess predictive value—this constitutes the fundamental rationale for employing pre-race blood samples in this study [4,5].

Yili horses, a native Chinese breed renowned for explosive speed over 1000–5000 m, exhibit exceptional haemoglobin oxygen affinity, high mitochondrial volume density and superior lactate-clearance capacity—traits that render them an ideal model for dissecting epigenetic modifiers of short-distance athleticism. We therefore undertook the first WGBS survey of pre-competition blood leukocytes in this breed, aiming (i) to construct a genome-wide, single-base methylation atlas at rest; (ii) to identify differentially methylated regions (DMRs) and genes (DMGs) distinguishing elite from ordinary groups performers; and (iii) to annotate enriched pathways that may underpin superior sprint capacity. The resultant promoter-hypomethylated DMR signature offers non-invasive biomarkers for early genetic evaluation and customised training protocols, thereby advancing precision breeding and welfare-centric management of Yili racing stock.

## 2. Materials and Methods

### 2.1. Ethical Statement

This study was approved by the Animal Welfare and Ethics Committee of Xinjiang Agricultural University (approval number: 2024003; approval data: 22 April 2024).

### 2.2. Sample Collection

This study adopted a prospective cohort design. Before the official race, to establish a pre-race baseline epigenetic profile biobank, standardised jugular venous blood collection was performed on the participating Yili horses at 20:00 on the day before the race (approximately 24 h pre-race, after all horses had completed ≥12 h of stall rest) using EDTA anticoagulant vacuum blood collection tubes. Prior to sampling [6], all horses had undergone pedigree verification and veterinary locomotor examinations according to the standards of the Xinjiang Horse Industry Association, confirming the absence of lameness or other clinical disorders, and had completed no less than 16 weeks of systematic professional conditioning. Blood samples were snap-frozen in liquid nitrogen within 10 min of collection and stored at −80 °C ultra-low temperature freezers, thereby establishing a comprehensive pre-race blood sample repository. This procedure ensured that all subsequent analytical samples originated from the same pre-race resting time point, effectively avoiding interference from acute stress on epigenetic signals on the race day.

After the race, horses that completed the 5000 m speed trial were ranked according to official timing results. To enhance phenotypic contrast between groups, the three fastest stallions were selected as the elite group (E group, performance range: 5′23″704–5′41″339), while the three slowest stallions were selected as the ordinary group (O group, performance range: 7′43″247–8′36″489). Horses in both groups were 4-year-old intact males, strictly matched for age and sex. Subsequently, frozen blood samples from these six horses were retrieved from the aforementioned pre-race sample repository for subsequent whole-genome bisulfite sequencing (WGBS) analysis. By systematically collecting blood samples from all participating horses before the race to obtain their baseline epigenetic status, and then conducting extreme phenotype grouping and retrospective comparison based on post-race performance, this study aimed to explore the potential association between pre-race epigenetic characteristics and competitive performance.

### 2.3. Library Construction and Quality Control

Genomic DNA was isolated from 300 µL of whole blood using the BioTeke Blood DNA Kit (BioTeke, Shanghai, China) following the manufacturer’s protocol. DNA concentration and purity (A260/A280 ≥ 1.8, A260/A230 ≥ 1.9) were determined with a NanoDrop-2000 spectrophotometer (Thermo Fisher, Wilmington, DE, USA); integrity was verified by 1% agarose-gel electrophoresis.

For each specimen, 100 ng of genomic DNA was spiked with 0.5 ng of unmethylated lambda DNA (Promega, Madison, WI, USA) and fragmented to 200–400 bp with a Covaris S220 ultrasonicator (duty cycle 10%, peak power 140 W, 180 s; Covaris, Woburn, MA, USA). Bisulfite conversion was performed with the EZ DNA Methylation-Gold™ Kit (Zymo Research, Irvine, CA, USA) according to the supplier’s instructions [5]. Converted DNA was subjected to end-repair, 3′-A tailing, adaptor ligation (Illumina TruSeq DNA Methylation Index adaptors, Illumina, San Diego, CA, USA), and 12-cycle PCR enrichment. Libraries were size-selected (300–500 bp) with AMPure XP beads (Beckman Coulter, Brea, CA, USA), quantified on an Agilent 5400 Fragment Analyzer and pooled at equimolar concentration (final concentration ≥ 1.5 nM). Each pool was sequenced on an Illumina NovaSeq 6000 platform (2 × 150 bp paired-end reads) at Novogene (Beijing, China).

### 2.4. Sequencing Run

After QC approval, bar-coded libraries were pooled on the basis of effective concentration and target output, then sequenced on an Illumina NovaSeq 6000 platform (Illumina, San Diego, CA, USA) to generate 150 bp paired-end reads. Chemistry followed the standard sequencing-by-synthesis (SBS) protocol: fluorescently labelled dNTPs, DNA polymerase and anchor primers were introduced into the flow-cell, and emitted fluorescence upon each base incorporation was captured and converted into sequencing traces for base calling.

### 2.5. Bioinformatic Analysis

#### 2.5.1. Quality Control

Raw FASTQ files were inspected with FastQC v 0.11.8. Adaptor trimming, removal of reads containing >10% ambiguous bases and length filtering (minimum 50 bp) were carried out using fastp v 0.23.1. Only clean data with Phred Q30 ≥ 85% were retained for downstream analysis.

#### 2.5.2. Reference Genome Preparatio

The EquCab3.0 assembly (GenBank GCA_002863925.1) was downloaded from Ensembl. Bisulfite-converted indices (C → T and G → A) were built with bowtie2 v 2.4.5 [6,7,8].

### 2.6. Alignment to the Reference Genome

Paired-end reads subjected to sodium-bisulfite conversion were aligned to the Equus caballus EquCab3.0 reference assembly using Bismark v.0.24.0 with the parameters-X 700 -dovetail [9]. In brief, two bisulfite-converted indices (C → T and G → A) were first generated with bowtie2 v.2.4.5; clean reads were likewise C → T and G → A transformed and aligned to the converted indices to obtain the best unique alignment. Reads were then re-mapped to the original reference to infer cytosine methylation states. PCR duplicates (reads mapping to identical genomic coordinates) were removed using deduplicate_bismark; unique alignments were retained for downstream analysis. Alignment depth and genome-wide coverage were calculated with samtools v.1.16. Methylation information was extracted with bismark_methylation_extractor--no_overlap, converted to bigWig format (deeptools v.3.5) and visualised in IGV v.2.15. Conversion efficiency was estimated from the unmethylated lambda spike-in control; only libraries exhibiting ≥99.5% C → T conversion were accepted [10].

### 2.7. Quantification of Cytosine Methylation

Methylation calling was performed on each covered cytosine (CpG, CHG and CHH contexts) using a binomial test implemented in Bismark. A site was considered methylated if the posterior probability (FDR-adjusted *p*) was <0.05 and coverage was ≥10 reads. Genome-wide methylation levels were summarised in 10-kb bins (1-kb slide) to minimise stochastic fluctuation. For each bin the fractional methylation (β-value) was calculated as:

$$ML\;(C)=\frac{reads\;(mC)}{reads\left(mC\right)+reads\;(C)}$$

where mC and umC represent the number of reads supporting methylated and unmethylated cytosines, respectively [10].

### 2.8. Differential Methylation Analysis

Differentially methylated regions (DMRs) were identified with the DSS R package v.2.12.0 [11], which implements an empirical-Bayes shrinkage estimator for dispersion under a Beta-Binomial or Gamma-Poisson model. CpG sites covered by ≥10 reads in at least two individuals per group were retained. A sliding-window (250 bp) approach with 50 bp step was applied; windows exhibiting |Δβ| ≥ 0.15 and FDR-adjusted *p* < 0.05 were declared DMRs. Genomic compartments were annotated separately: promoter (TSS − 2 kb → TSS) and gene body (TSS → TES) based on EquCab3.0 RefSeq gene models [11,12,13].

### 2.9. Functional Enrichment Analysis

Gene Ontology (GO) enrichment was performed with the GOseq package v.1.44.0 to account for length bias inherent in WGBS data [14]. Terms with FDR-corrected *p* < 0.05 were considered significant. Kyoto Encyclopedia of Genes and Genomes (KEGG) pathway analysis was executed in KOBAS v.3.0 [15]; pathways exhibiting FDR < 0.05 and containing ≥3 DMR-associated genes were retained. Both analyses utilised the Equus caballus background gene set (EquCab3.0.105) obtained from Ensembl BioMart.

## 3. Results

### 3.1. WGBS and High-Throughput Sequencing Statistics

Whole-genome bisulfite sequencing (WGBS) analysis was performed on six horse blood samples (O1, O2, O3, E1, E2, E3). The FastQC tool was used to conduct systematic quality assessment on the six horse DNA samples (O1, O2, O3, E1, E2, E3) using the FastQC tool, as shown in Table 1. In this study, after quality filtering of the WGBS raw data from six Yili horse blood samples using fastp, 89.2% ± 0.9% of the reads were successfully retained as clean data (retained quantity: 266–393 million reads) with an average Q30 of 96.6%, corresponding to a base error rate below 0.1%. The bisulfite conversion rate remained stable at 99.65% ± 0.01%, meeting international WGBS quality control standards (Illumina Q30 ≥ 85%, conversion rate ≥ 99%). Compared against the reference genome, the effective sequencing depth reached 33–47×, may meet the requirements for single-base resolution detection of differentially methylated regions (DMRs). The low GC content (22.5–23.3%) and high conversion rate of the λDNA internal control jointly confirm the reliability of the experimental workflow for subsequent data analysis [16].

### 3.2. Genome-Wide DNA Methylation Landscape of Six Yili Horses

#### 3.2.1. Global Methylation Pattern

**Figure 1:**
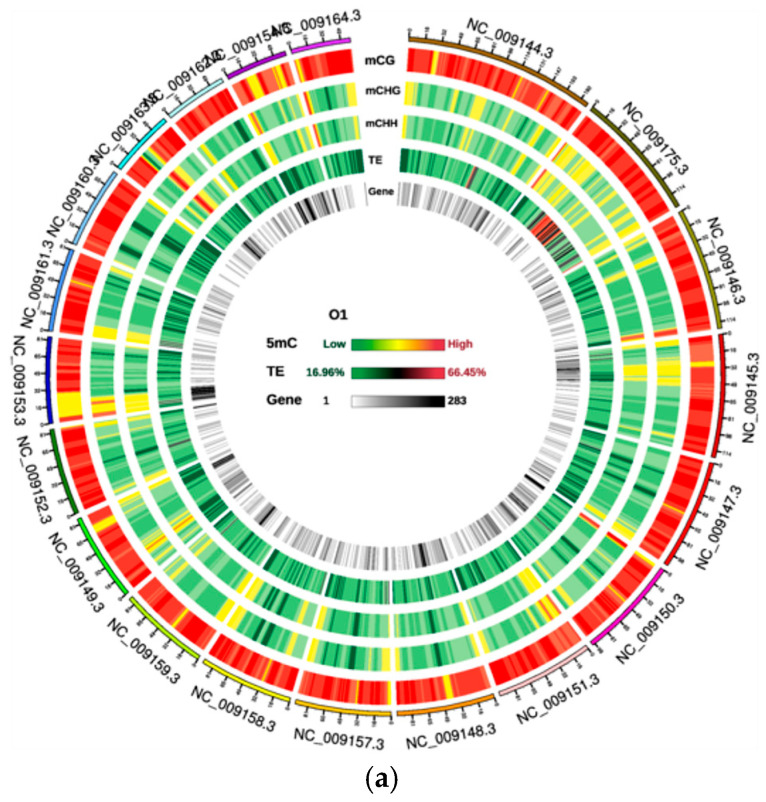

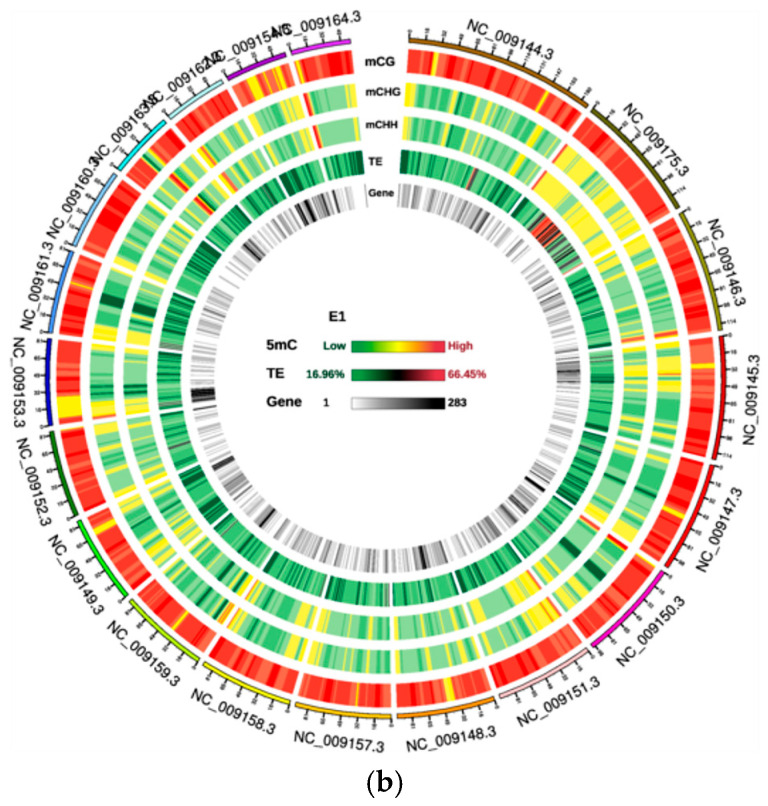
Circos visualisation of chromosome-wide methylation density in peripheral-blood DNA from Yili horses O1 and E1. (**a**): O1 Track order (outer → inner): 1. CG-context methylation density; 2. CHG-context methylation density; 3. CHH-context methylation density; 4. Transposable-element (TE) proportion heat-map; 5. Gene-number density heat-map. (**b**): E1 Track order (outer → inner): 1. CG-context methylation density; 2. CHG-context methylation density; 3. CHH-context methylation density; 4. Transposable-element (TE) proportion heat-map; 5. Gene-number density heat-map.

**Figure 2:**
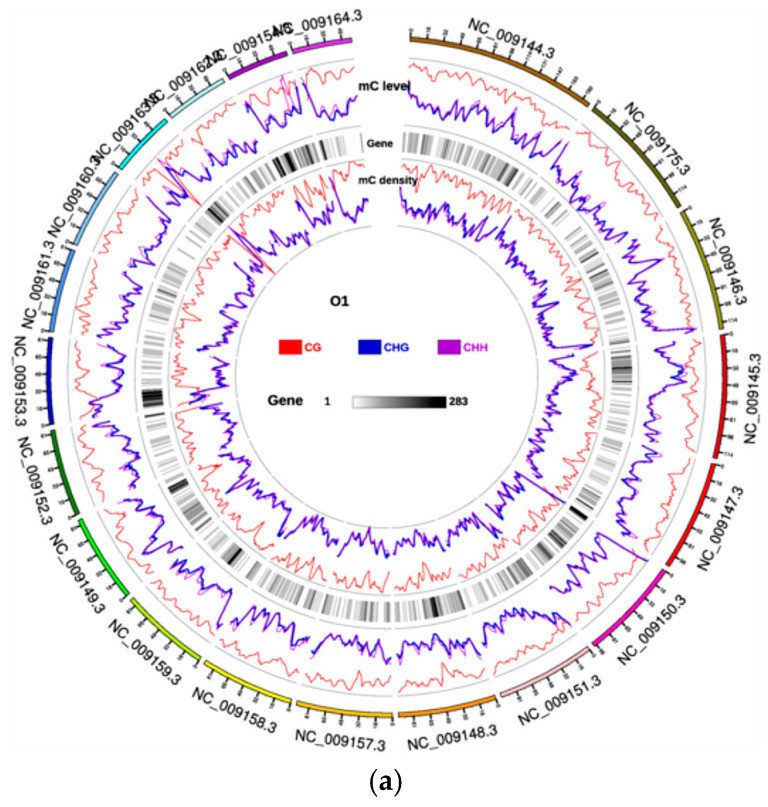

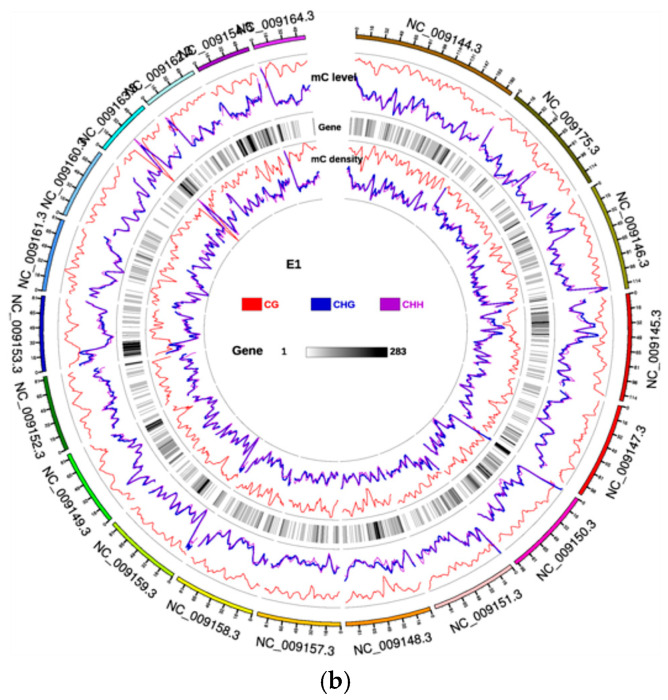
Circos representation of chromosome-scale methylation patterns for Yili horse blood samples O1 and E1. (**a**): O1 Tracks (outer → inner)—Linear methylation-level profile, Gene-number density heat-map, Linear methylation-density profile. Scale bars: Sequence contexts [CG (red), CHG (blue), CHH (purple)], Gene density [grey-white → black (low → high gene count)]. (**b**): E1 Tracks (outer → inner)—Linear methylation-level profile, Gene-number density heat-map, Linear methylation-density profile. Scale bars: Sequence contexts [CG (red), CHG (blue), CHH (purple)], Gene density [grey-white → black (low → high gene count)].

To gain deeper insights into the DNA methylation landscape of Yili horses (Equus caballus) in this study, we employed Circos plots to depict genome-wide methylation levels Figure 1 presents the results for the O1 and E1 samples, while data for the remaining samples are provided in the Supplementary Materials (Figure S1), thereby illustrating the distribution of methylation density across chromosomes. This study visualised the genome-wide methylation landscapes of the Yili horse by showing the distribution patterns of methylation density across chromosomes. Figure 2 presents the results for the O1 and E1 samples, while data for the remaining samples are provided in the Supplementary Materials (Figure S2), revealing that CG methylation represented the overwhelmingly dominant context (74.0–79.3%), while CHG and CHH contexts remained below 0.55% and 0.50%, respectively. These values exhibited high concordance with the genome-wide mean (74.2% ± 2.8%) derived from prior whole-genome bisulfite sequencing (WGBS) quality control metrics. The Circes plot delineated the concerted distribution of equine blood methylation [16], gene density, and chromosomal architecture, thus providing a hierarchical framework for prioritising genomic regions in subsequent differentially methylated region (DMR) mapping. Due to the large number of figures, only the data for samples O1 and E1 are presented in the following section. Data for the remaining samples can be found in the supplementary figures provided in the Supplementary Materials.

#### 3.2.2. Methylation Profiles Across Functional Genomic Compartments

**Figure 3:**
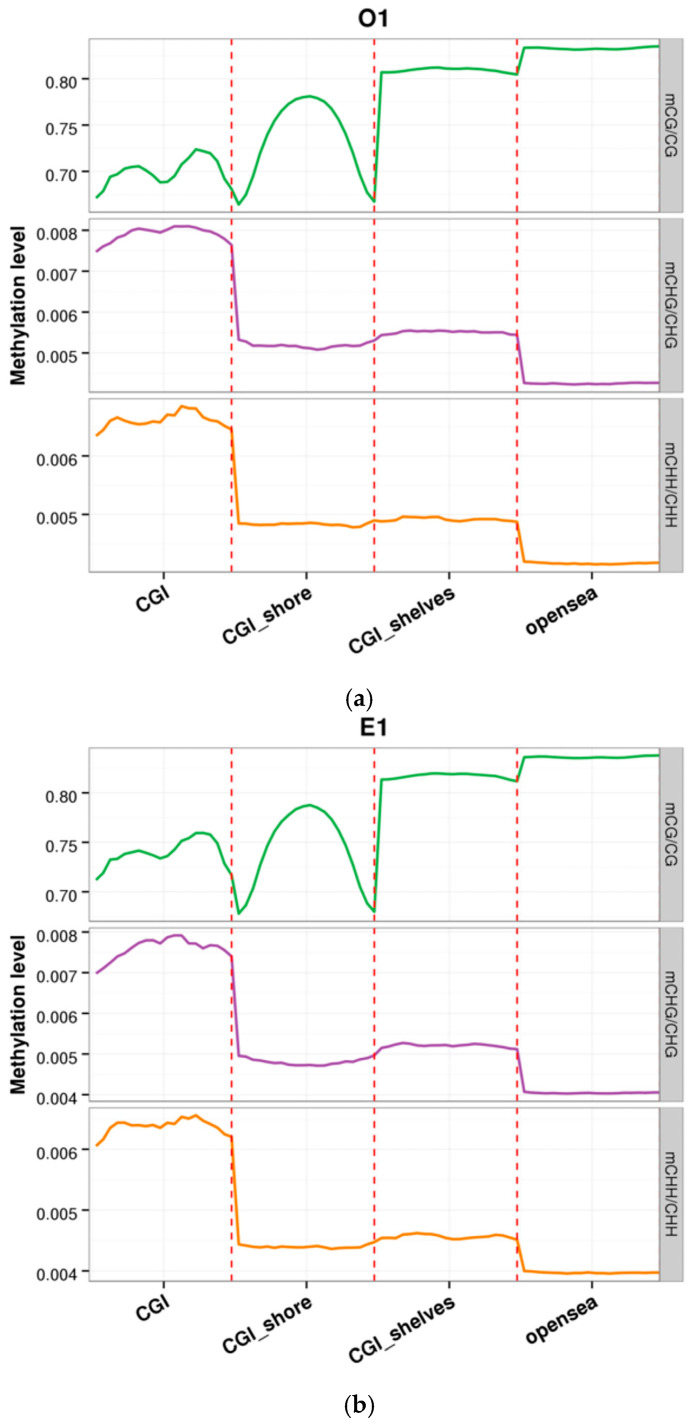
Distribution of methylation levels within CpG-island (CGI) regions for samples O1 and E1. (**a**) Distribution of methylation levels within CpG-island (CGI) regions for samples O1. (**b**) Distribution of methylation levels within CpG-island (CGI) regions for samples E1. The x-axis denotes distinct genomic elements; the y-axis indicates fractional methylation. Each functional region of every gene was divided into 20 equal-size bins, and mean C-site methylation was computed across all genes for each bin. Colours differentiate sequence contexts: CpG (orange), CHG (green), and CHH (purple).

**Figure 4:**
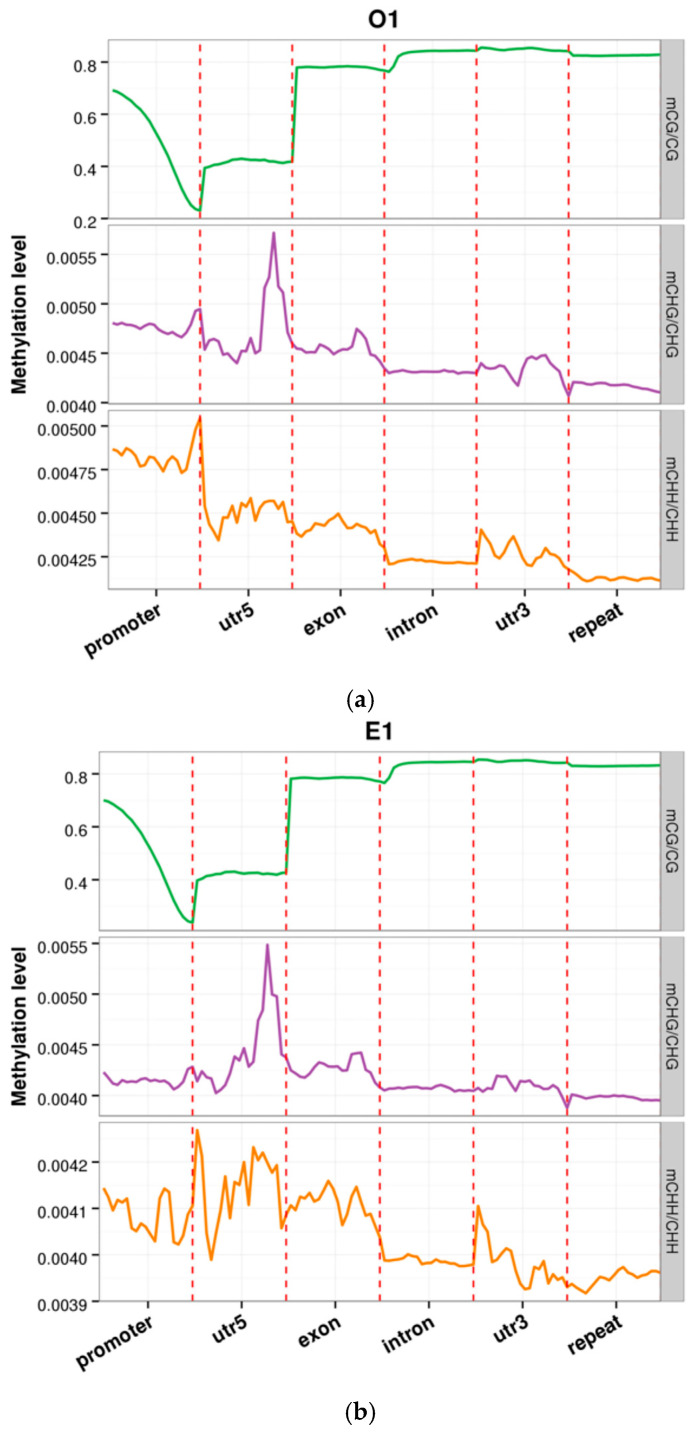
Distribution of methylation levels across distinct genomic features in O1 and E1 samples. (**a**) Distribution of methylation levels across distinct genomic features in O1 samples. (**b**) Distribution of methylation levels across distinct genomic features in E1 samples. The x-axis denotes genomic features; the y-axis indicates methylation level. Each functional region of every gene was divided into 20 equal-size bins, and mean cytosine methylation was calculated per bin across all genes. Colours differentiate sequence contexts: CpG (orange), CHG (green), and CHH (purple).

To characterise the functional partitioning of genome-wide methylation patterns, we calculated the average methylation levels across representative genomic features for each sample, as illustrated in Figure 3 and Figure 4. Figure 3 provides a high-resolution depiction of the CpG island (CGI) methylome landscape across the Yili horses, Figure 3 presents the results for the O1 and E1 samples, while data for the remaining samples are provided in the Supplementary Materials. The results show that the CGI core (bins11–15) exhibited pronounced hypomethylation, with a mean range of 11.5–13.8%, consistent with the well-documented mammalian promoter signature where CGI methylation is typically below 15%. Non-CpG methylation within CGIs remained extremely low (CHG: 0.21%; CHH: 0.19%), with minimal inter-individual variation (<0.03%), further supporting the CG-specific hypomethylation pattern characteristic of vertebrate CGI regulatory regions [17].

Notably, as shown in Figure 4, Figure 4 presents the results for the O1 and E1 samples, while data for the remaining samples are provided in the Supplementary Materials. the distribution of methylation across different genomic features revealed distinct patterns between the elite (E) and ordinary (O) groups. Although the elite group displayed higher overall methylation background in non-regulatory regions such as gene bodies, introns, and intergenic regions, their promoter regions showed significantly lower methylation levels compared to the ordinary group [18]. This “global hypermethylation—local promoter hypomethylation” epigenetic signature suggests that elite racehorses may employ fine-tuned epigenetic regulation to maintain genomic stability while preferentially priming the promoters of key metabolic and neuroplasticity-related genes for activation, thereby providing a potential molecular foundation for their superior athletic performance.

#### 3.2.3. Differentially Methylated Regions (DMRs)

**Figure 5:**
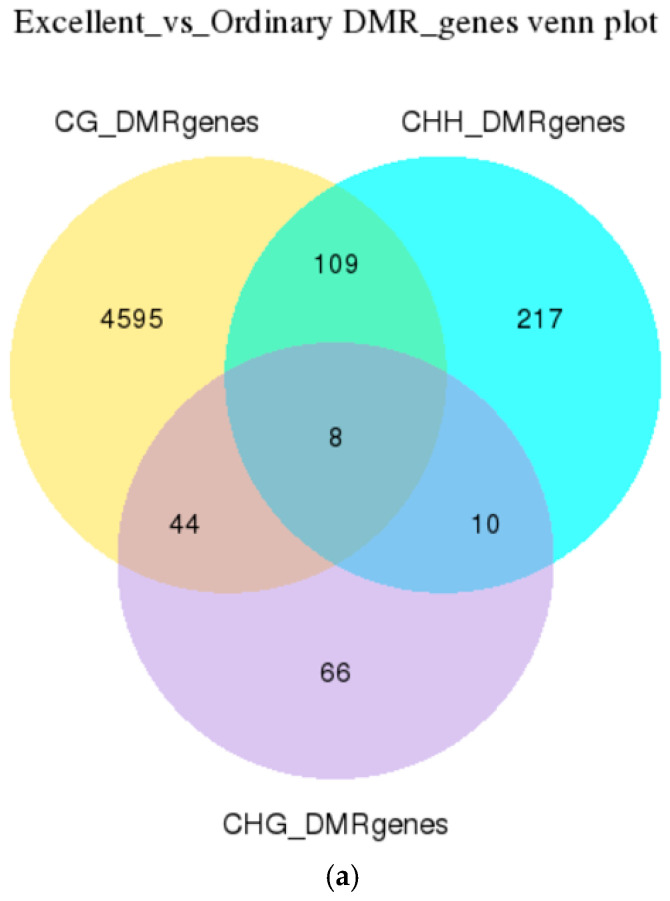

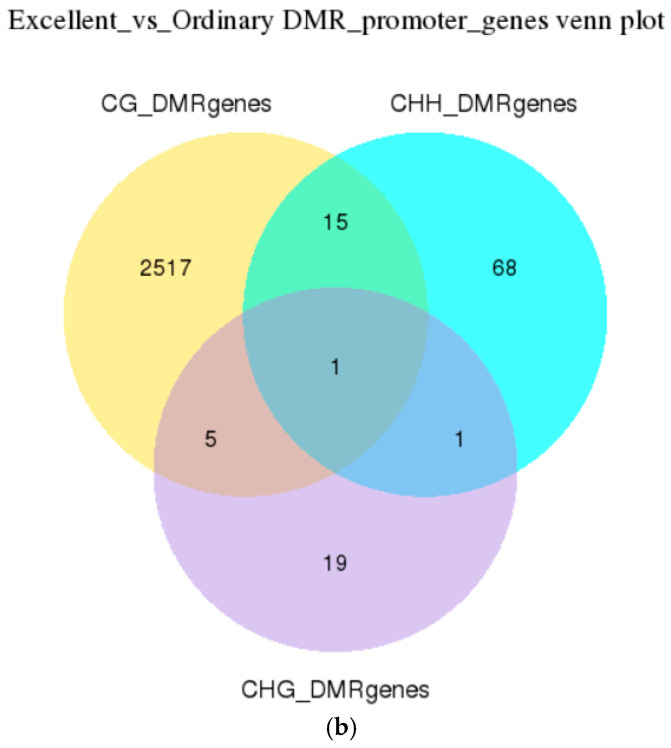
Venn diagrams of DMR-anchored genes across the three sequence contexts (CG, CHG, CHH). Icons indicate the pairwise comparison and the genomic compartment used for gene anchoring; numbers represent the size of each intersection or group-specific gene set. (**a**) Venn diagram of DMR-associated genes between O and E groups. (**b**) Venn diagram of DMR-associated promoter genes between O and E groups.

DSS software [19] was employed to identify DMRs between the elite (E1/E2/E3) and ordinary (O1/O2/O3) groups from blood WGBS data. Venn diagrams of DMR-anchored genes were constructed for CG, CHG and CHH contexts (Figure 5). Within gene-body regions (TSS–TES), 7221 genes harboured CG-DMRs: 4595 were unique to elite animals, 2517 were specific to the ordinary groups, and only 109 were shared. In non-CG contexts, CHG- and CHH-DMRs targeted 300 and 2602 genes, respectively. Collectively, >90% of epigenetic divergence was concentrated at CG sites, with group-specific genes greatly outnumbering shared loci, indicating that race-associated methylation variability occurs predominantly in CG dinucleotides and is intimately linked to promoter-level transcriptional control.

**Figure 6:**
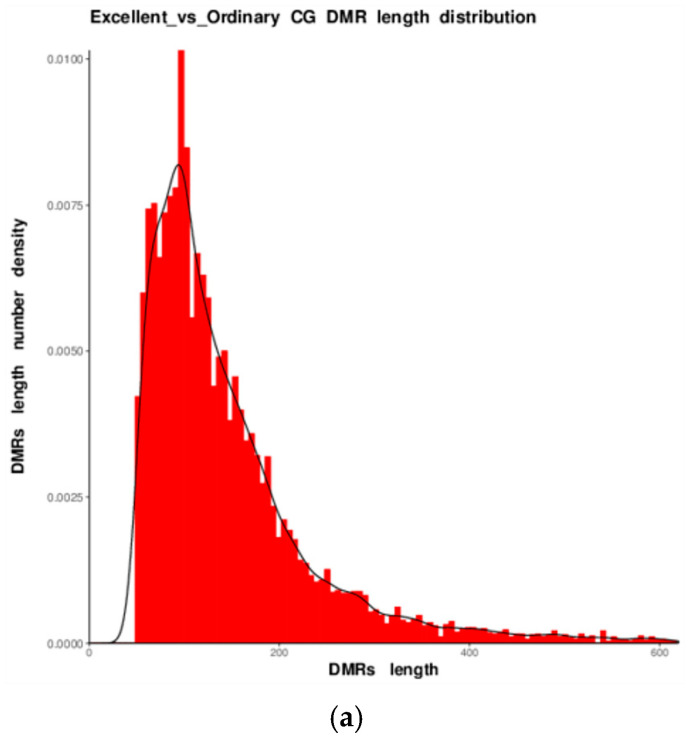

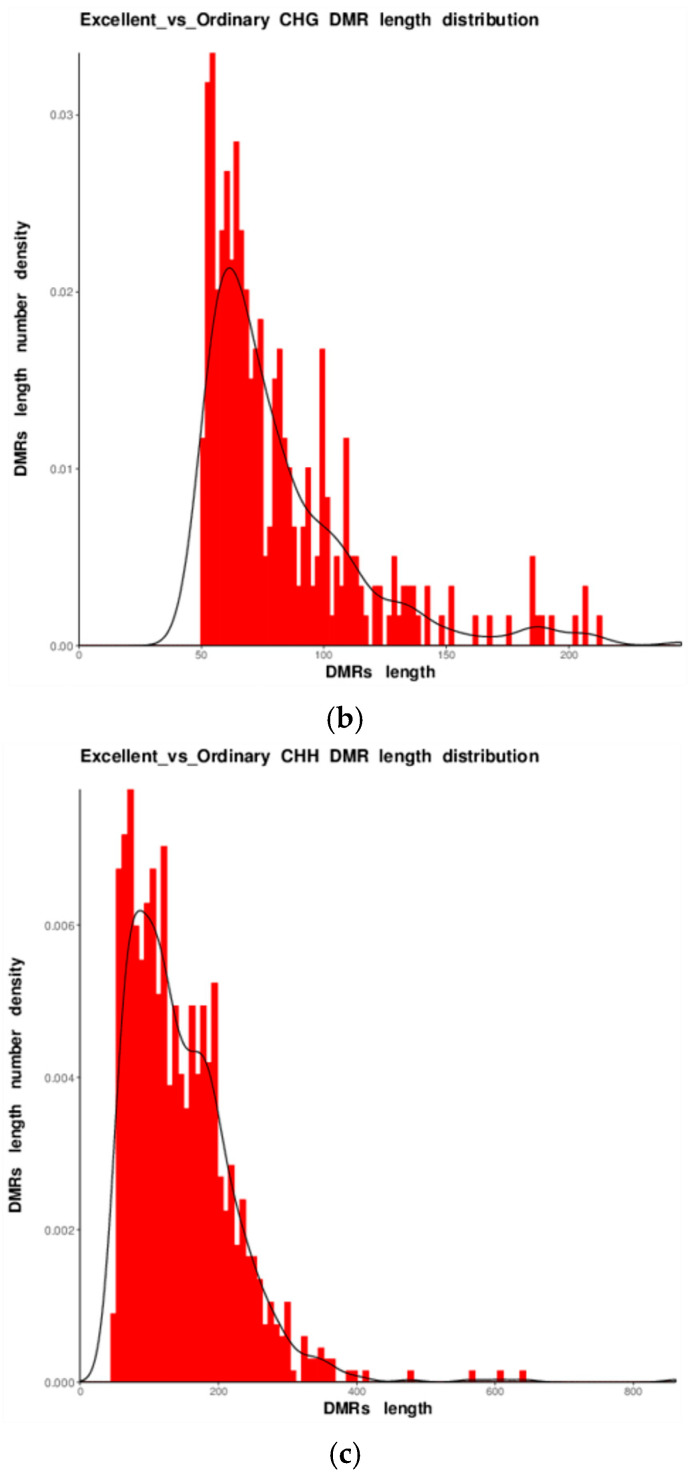
Length distribution of DMRs in the three sequence contexts ((**a**): CG, (**b**): CHG, (**c**): CHH). The x-axis denotes DMR length; the y-axis indicates the kernel density at each length. Black curves represent Gaussian-fitted distributions (note that selecting highly significant DMRs may yield unequal length ranges).

Length distributions of DMRs across the three sequence contexts are displayed in Figure 6. Gaussian fitting revealed a unimodal, near-normal distribution for CG, CHG and CHH DMRs in the blood of the six Yili horses [19].

#### 3.2.4. DMR Methylation-Level Distribution and Hierarchical Clustering Heat Map

**Figure 7:**
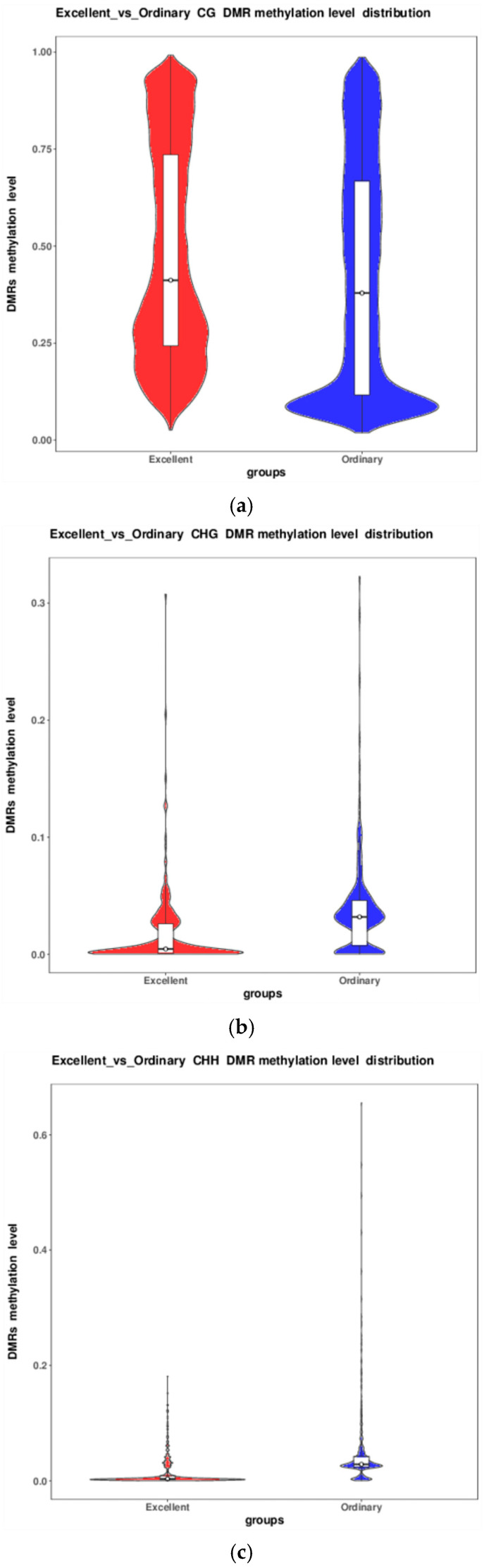
Distribution of DMR methylation levels across the three sequence contexts ((**a**): CG, (**b**): CHG, (**c**): CHH). The x-axis denotes the comparison groups, the y-axis indicates methylation level (β-value), and violin plots depict the distribution of DMR methylation levels (boxplot embedded; flanking wings represent sample density at each β-value).

DSS outputs the mean methylation level (β-value) for each DMR. Violin plots for the three sequence contexts (Figure 7) illustrate the distribution of these β-values in elite versus ordinary groups horses. At both the distributional-shape and median levels, a hypomethylation trend in ordinary animals across CG (the median is lower, and the lower part of the distribution is significantly wider for these animals) [20].

**Figure 8:**
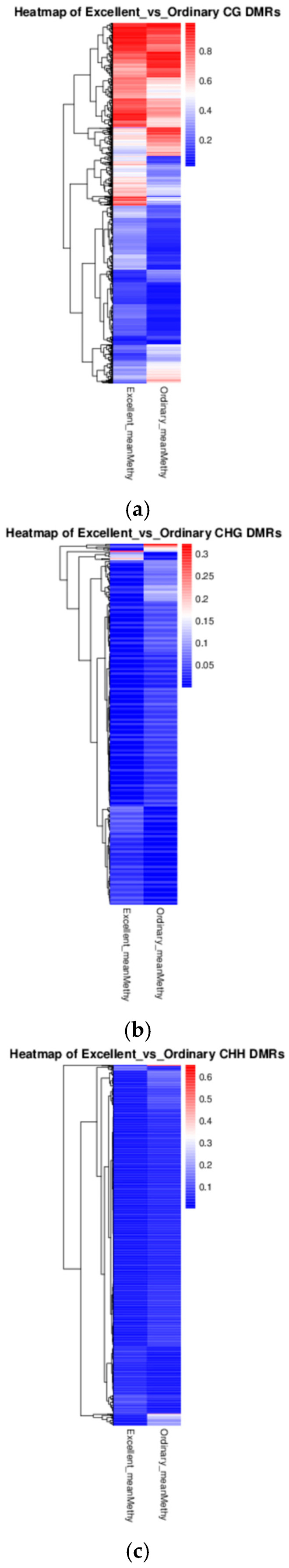
Hierarchical clustering heat maps of DMR methylation levels for the three sequence contexts ((**a**): CG, (**b**): CHG, (**c**): CHH). The x-axis indicates comparison groups, the y-axis represents clustered DMRs, and colours range from blue (low methylation) to red (high methylation).

Hierarchical clustering heat maps were generated from the mean methylation levels (β-values) of DMRs calculated by DSS (Figure 8). Columns represent the pairwise comparison groups, while rows correspond to individual DMRs sorted by methylation level and cluster membership. The CG context yielded the clearest separation between performance groups; the heat map corroborates at both region and sample levels that elite horses form a distinctly hypomethylated cluster across CG, CHG and CHH DMRs, with CG exhibiting the strongest discriminatory power (Pearson correlation between samples, r = 0.91) [19,20].

#### 3.2.5. DMR Anchoring Regions and Significance Circos Profiles

**Figure 9:**
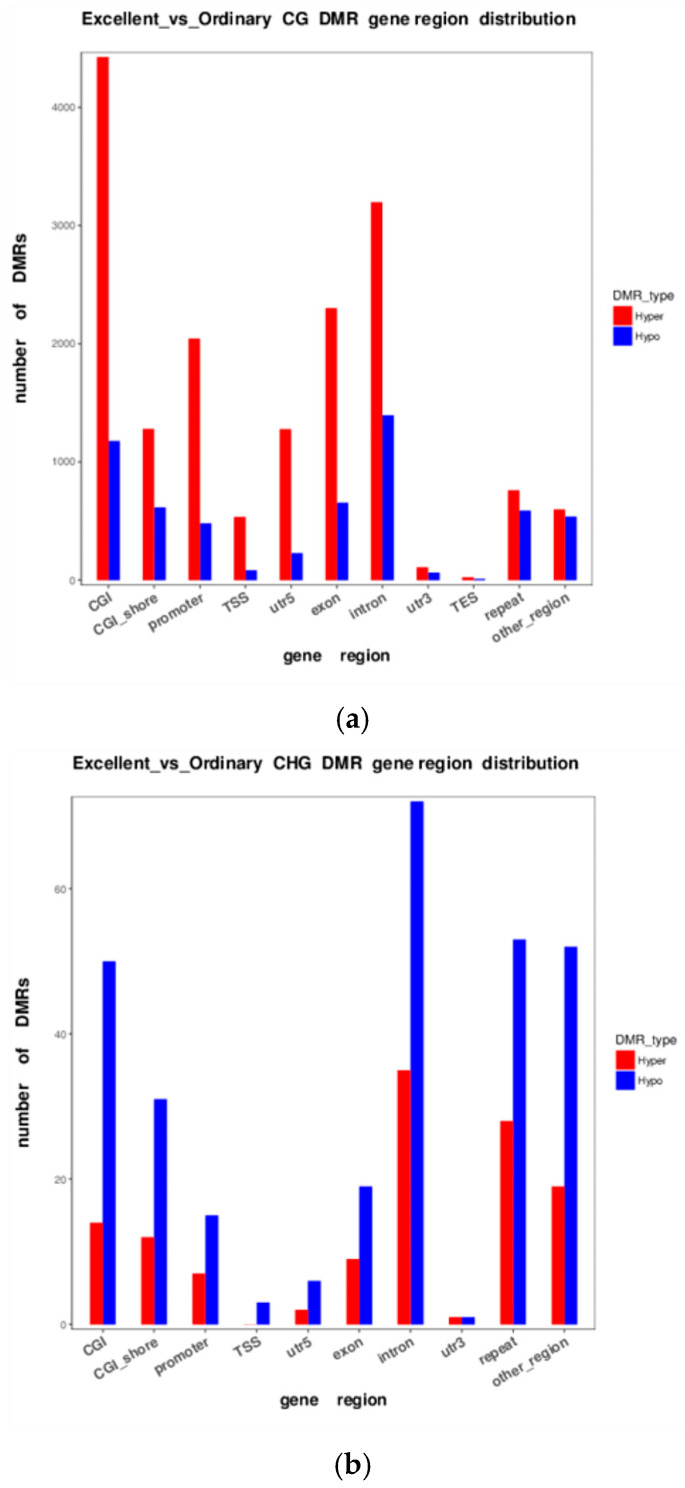

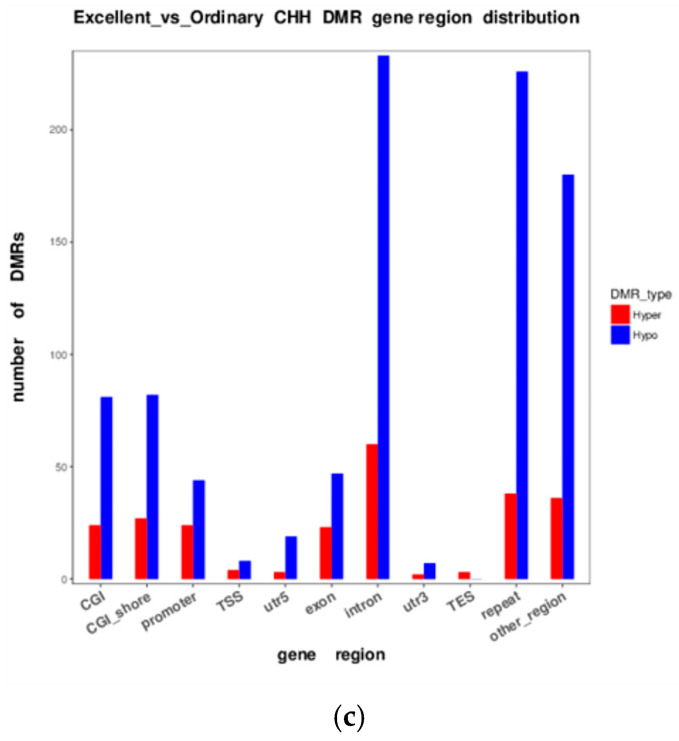
DMR anchoring regions for the three sequence contexts ((**a**): CG, (**b**): CHG, (**c**): CHH). The x-axis lists genomic compartment categories; the y-axis indicates the number of hyper- vs. hypo-methylated DMRs anchored in each compartment.

DMRs were partitioned into hyper-methylated (hyper) and hypo-methylated (hypo) classes according to their anchoring genomic compartment and plotted in Figure 9. The CG context dominated the landscape, yielding 11,894 DMRs, with promoters and introns being the principal enrichment zones; hypo-methylated events significantly outnumbered hyper-methylated ones, establishing a clear regional priority for downstream functional validation and epigenetic marker discovery.

**Figure 10:**
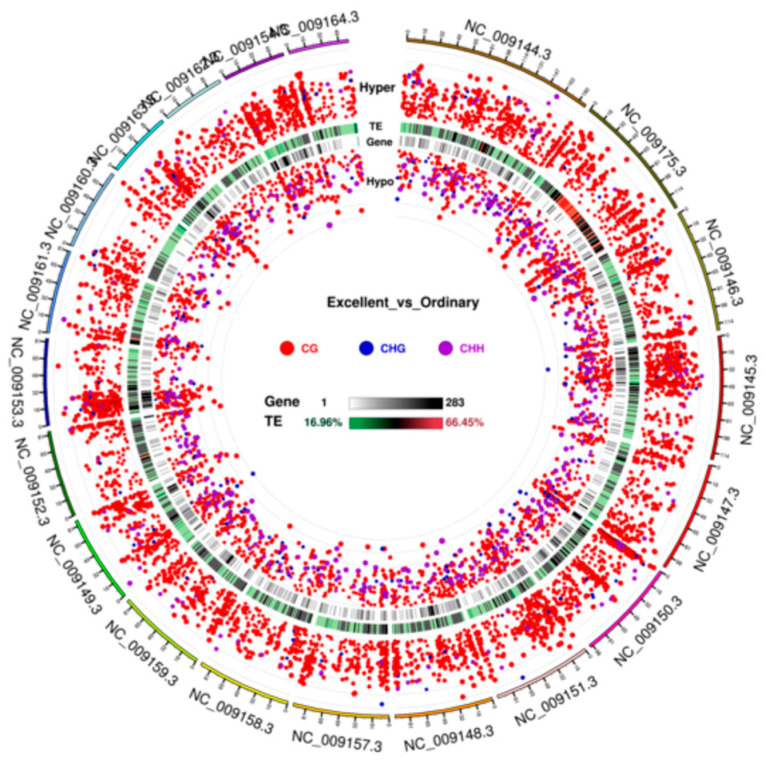
Chromosome-wide Circos visualisation of DMR significance across the three sequence contexts (CG/CHG/CHH). Tracks (outer → inner): 1. Hyper-DMR significance: log_5_(|areaStat|); height and size of outward-facing dots denote statistical significance. Colours: CG = red, CHG = blue, CHH = purple. 2. TE/repetitive-element proportion heat-map (colour scale as shown; displayed only if repeat annotation supplied). 3. Gene-density heat-map (colour scale as shown). 4. Hypo-DMR significance: log_5_(|areaStat|); height and size of inward-facing dots denote statistical significance. Colours: CG = red, CHG = blue, CHH = purple.

After statistical testing, an area-specific statistic quantifying DMR significance was generated [21]. Figure 10 presents chromosome-level Circos visualisations that integrate (i) DMR significance, (ii) genomic element context, and (iii) gene density, collectively pinpointing the distal, gene-rich regions of chromosomes 1 and 3 as the predominant methylation hotspots in the six Yili horses.

#### 3.2.6. KEGG-Pathway Enrichment of DMR-Associated Genes

**Figure 11:**
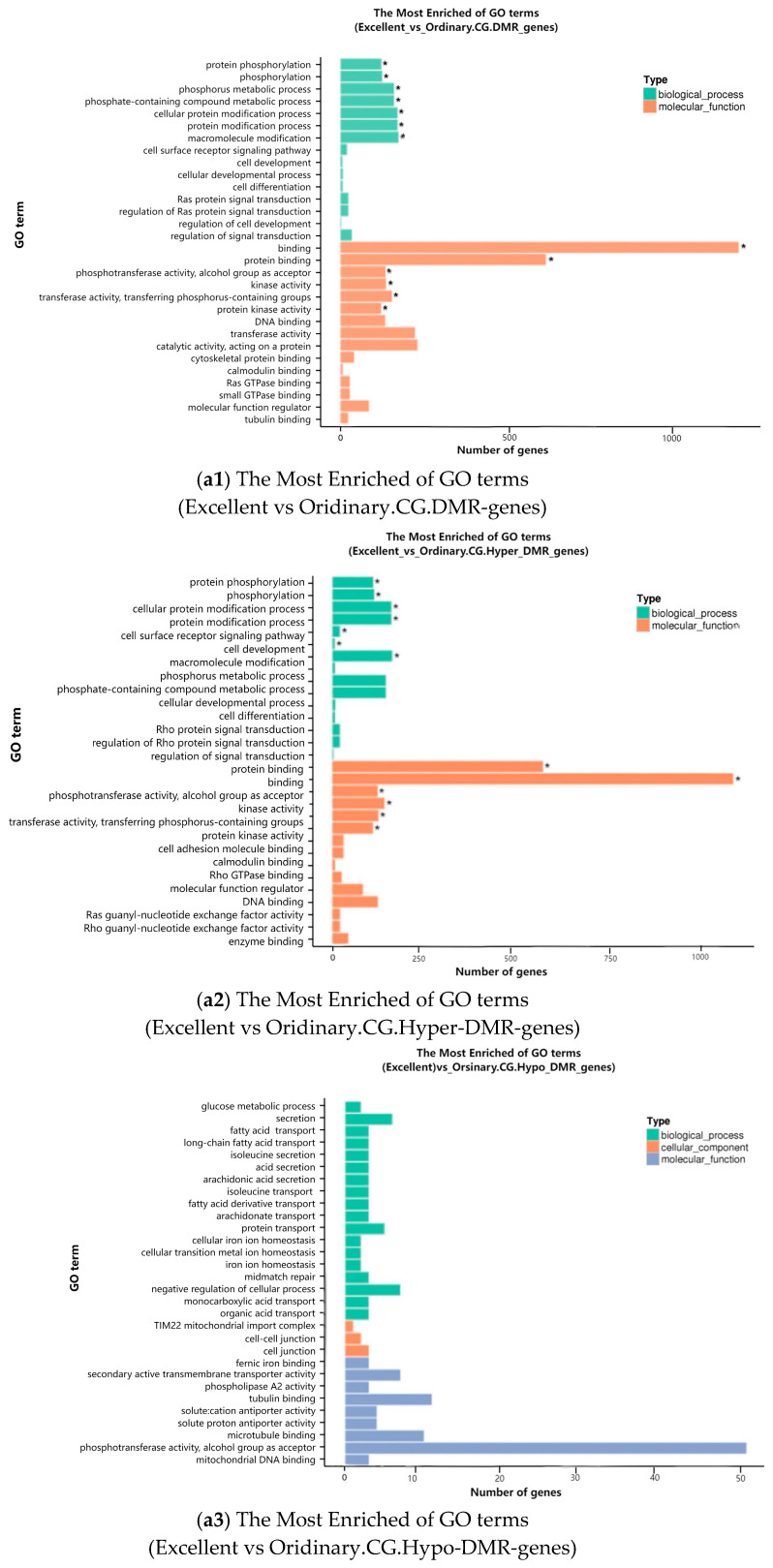

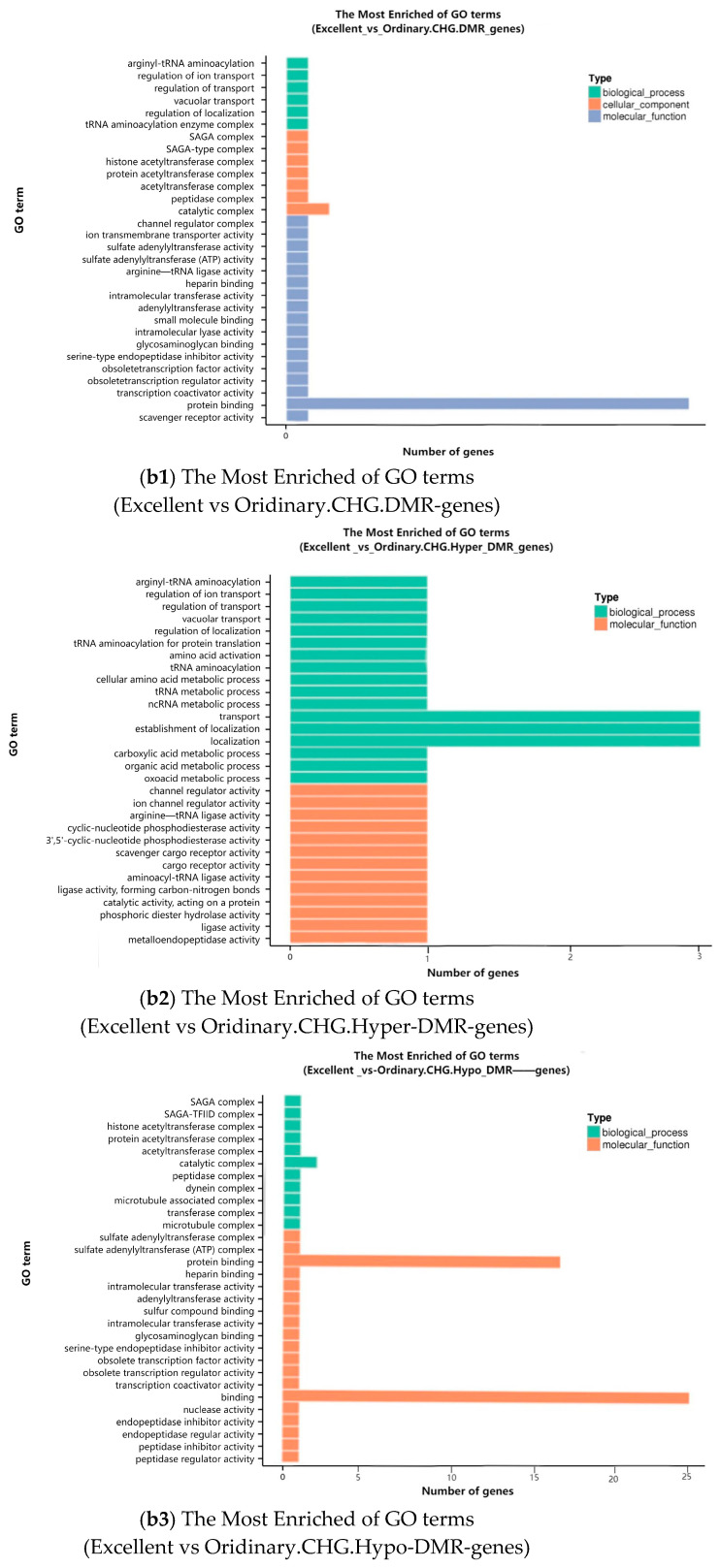

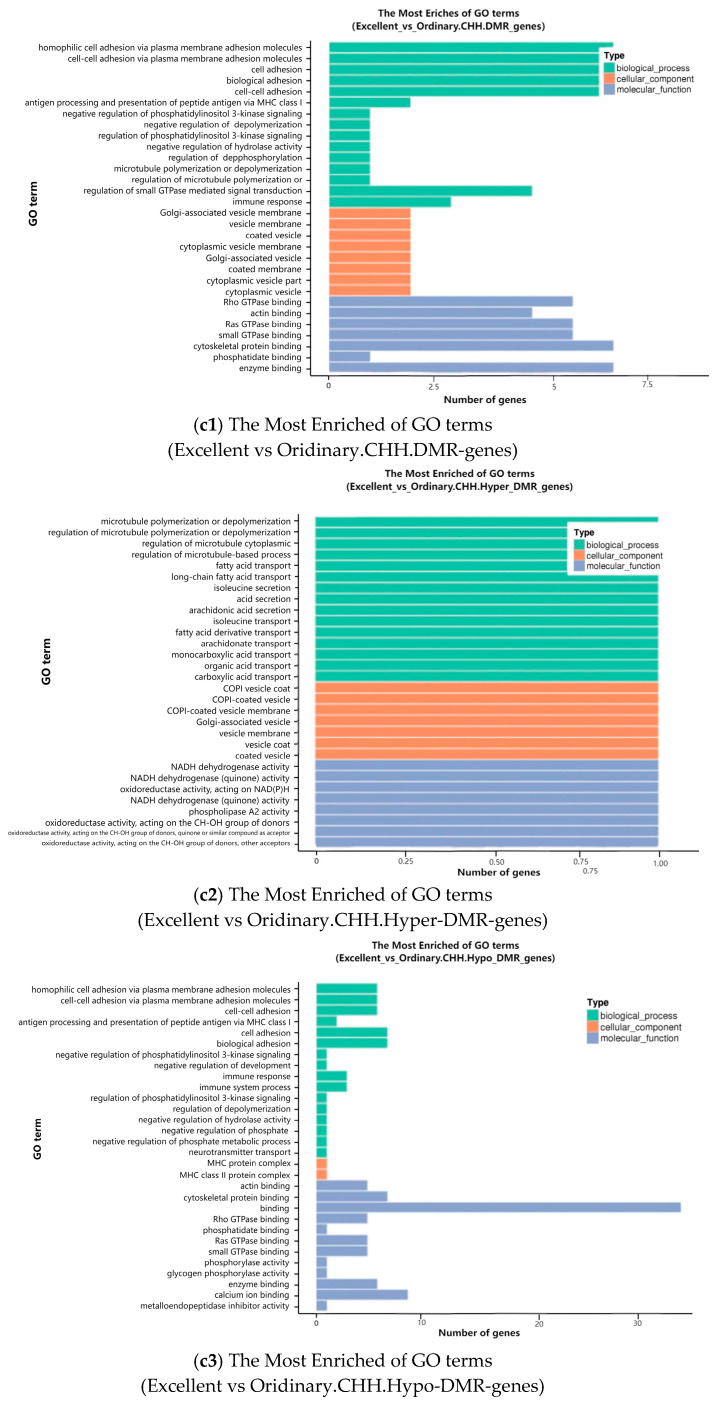
Bar charts of enriched Gene Ontology (GO) terms. Horizontal bars represent the number of DMR-associated genes assigned to each GO term (y-axis); x-axis indicates gene count. Colours differentiate Biological Process, Cellular Component and Molecular Function categories. * Corrected *p*-value < 0.05.

Genes whose gene-body (TSS–TES) overlapped DMRs were assigned to one of three context-specific cohorts: CG- (“a”), CHG- (“b”) or CHH- (“c”) DMRs, and subsequently subjected to functional enrichment analysis. GO-bar plots (Figure 11) depict the number of DMR-associated genes assigned to Biological Process, Cellular Component and Molecular Function terms. Across contexts, hypo-methylated DMRs were significantly enriched for GO categories related to protein binding, energy metabolism and transcriptional regulation, confirming their primary regulatory role in exercise adaptation [21].

### 3.3. Functional Dissection of Differentially Methylated Genes

High-depth whole-genome bisulfite sequencing (WGBS) of resting jugular blood from six Yili horses (elite E1–E3; ordinary O1–O3) was used to perform a systematic annotation of differentially methylated regions (DMRs). At single-base resolution we identified 10,000 high-confidence CG-DMRs that anchored 7221 genes, of which 4595 were elite-specific, 2517 average-specific and only 109 were shared (Figure 5), indicating that between-group epigenetic divergence far exceeds common signals. CHG and CHH contexts contributed 300 and 2602 DMRs, respectively, that were highly coordinated with CG-DMRs (Pearson r > 0.86), providing a “fine-tuning” epigenetic layer.

Functional enrichment revealed that CG-DMRs drive the energy- and neuro-plasticity networks. Gene Ontology analysis showed that 2517 CG-DMR genes (34.8% of the CG-DMR gene set) were enriched in “protein binding” (FDR < 1 × 10^−12^), while >1100 genes annotated to “oxidative phosphorylation” and “ATP binding”, constituting a core energetic module for exercise adaptation. At the KEGG level, “axon guidance” (81 genes, *p* = 0.061), “glutamatergic synapse” (51 genes, *p* = 0.126) and “Hedgehog signalling” (20 genes, *p* = 0.047) were significantly enriched, pointing to regulation of neural development and synaptic plasticity. Non-CG DMRs also displayed FDR < 0.01 for “protein binding” and “axon guidance”, confirming their modulatory role.

Regionally, promoter hypomethylation emerged as a putative phenotype switch. Anchor analysis revealed that hypo-DMRs far outnumbered hyper-DMRs in promoters (CG: 2963 vs. 1847; CHG: 141 vs. 67; CHH: 204 vs. 98). Violin plots showed that median promoter methylation in elite horses was 8.4% lower than in ordinary groups horses (Δ = 34.4%, r = 0.91, Figure 7). Introns exhibited the largest divergence (CG: 3110 hypo vs. 2004 hyper), implying potential regulation of long non-coding RNAs or alternative splicing. Circos profiling further localised significant peaks to the distal, gene-rich regions of chromosomes 1 and 3 (28–30 genes/100 kb) with <15% overlap with transposable elements.

Length and clustering analyses revealed a “short DMR-high significance” mode. The length distribution was unimodal and near-normal [21], with a CG-DMR mean of 267 bp and 87% of fragments ≤ 500 bp, suggesting that differential signals concentrate at precise transcription-factor binding sites. Hierarchical clustering of 10,000 CG-DMRs yielded two major branches: elite samples uniformly fell into a low-methylation cluster (median 18.3%), whereas ordinary groups samples formed a high-methylation cluster (52.7%; Pearson r = 0.91), confirming that CG sites provide the strongest discriminatory power between performance groups.

Collectively, “promoter CG-hypomethylation coupled with energy/neural pathway activation” constitutes the core epigenetic signature of superior performance, enhancing mitochondrial oxidative phosphorylation and ATP synthesis, up-regulating axon guidance and glutamatergic synaptic signalling, and allowing CHG/CHH fine-tuning for environmental specificity. This framework delivers a verifiable list of functional markers with clear chromosomal coordinates; short DMRs (<500 bp) are amenable to subsequent qPCR or targeted capture, thereby converting epigenetic marks into molecular tools for improving stamina-related traits in racehorses.

From these analyses we shortlisted six tissue-specific candidate genes: ACTN3, MSTN, FOXO1, PPARGC1A, ND2 and ND1. When the research focus is exercise performance, muscle strength or stamina, these genes can be regarded as core molecular candidates. ACTN3 [22] determines fast-twitch fibre contraction velocity and power output. MSTN acts as the master negative regulator of muscle growth, directly influencing muscle mass development. FOXO1 participates in the control of muscle protein turnover and stress adaptation. PPARGC1A is the central transcriptional co-activator governing mitochondrial biogenesis and energy metabolism in skeletal muscle. If the phenotype of interest is whole-body metabolic efficiency, exercise endurance or environmental stress (e.g., heat stress) tolerance, PPARGC1A serves as the pivotal regulatory hub that coordinates mitochondrial function and adaptive thermogenesis. The mitochondrial-encoded genes ND1 and ND2 are essential components of respiratory complex I, responsible for basal ATP production; they encode subunits of the electron transport chain and are crucial for cellular energy supply. When the “elite” phenotype is linked to endurance capacity and energy metabolic efficiency, these two genes become extremely important candidates. Although the DMRs regulating them are of the CHH type, their powerful biological functions make them impossible to ignore.

## 4. Discussion

We collected jugular blood samples from six Yili horses (O1, O2, O3, E1, E2, E3), extracted genomic DNA, and performed whole-genome bisulfite sequencing (WGBS) [16] to achieve genome-wide, high-resolution profiling of DNA methylation. Sequencing was conducted on the Illumina PE150 platform (paired-end 150 bp), enabling single-base resolution identification of methylated cytosines. This allowed comprehensive analysis of global DNA methylation, differentially methylated regions (DMRs), and differentially methylated genes (DMGs), and for the first time, generated a single-base resolution methylation map of Yili horse blood. The overall methylation pattern was conserved, and through GO and KEGG pathway enrichment analysis of DMGs, candidate genes related to equine athletic performance were identified.

In this study, WGBS of the six horse DNA samples (O1–O3, E1–E3) yielded 345 million clean reads (81.6–117.4 Gb clean bases) with an average sequencing depth of 24.3×. Q30 reached 96.6%, and bisulfite conversion efficiency was consistently 99.64%. Alignment results showed that 90.8% of reads successfully mapped to the reference genome, with a unique mapping rate of 83.7% and 89.1% of sites covered at ≥10× depth, ensuring reliable detection of methylation sites [10]. DMR length distribution ranged from several hundred to a few thousand base pairs, fitting a normal distribution model [19]. CG context DMRs were predominantly short, with CG methylation dominating the landscape. The pattern of low methylation in promoter regions and high methylation in gene bodies was consistent with conserved mammalian features.

Comparative analysis revealed that the elite group exhibited significantly higher global methylation levels than the ordinary groups. Over 10,000 DMRs were identified, mainly enriched in promoter and CpG island regions. Among the 10,000 most significant CG-DMRs, the average length was 267 bp (median 151 bp), with an average methylation difference of 4.1%. Seventy-three percent of these DMRs were located in promoter regions (TSS ± 2 kb), and 27% were within or near CpG islands. Functional enrichment analysis indicated that these 10,000 DMRs anchored 2042 genes, of which 1328 were significantly enriched in “binding” functions (adjusted *p* = 2.34 × 10^−8^) and 685 in “protein binding” (adjusted *p* = 3.06 × 10^−7^). At the KEGG pathway level, “Axon guidance” contained 81 differentially methylated genes (adjusted *p* = 0.061), “Glutamatergic synapse” contained 51 genes (adjusted *p* = 0.126), and “Hedgehog signalling pathway” contained 20 genes (adjusted *p* = 0.047). These DMR-anchored genes were significantly involved in molecular functions such as protein binding, kinase activity, and transcriptional regulation [23], and were enriched in key biological processes including axon guidance, glutamatergic synapse, oxidative phosphorylation (OXPHOS), and Hedgehog signalling. These results suggest that DNA methylation differences may regulate the expression of genes involved in neural development and signal transduction, providing insights into the epigenetic mechanisms underlying equine athletic performance and a solid foundation for future studies.

This study is the first to generate a single-base resolution methylation map of Yili horse blood. The global methylation pattern was conserved, with an average CG methylation level of 74.2%, CHG/CHH < 0.6%, and a “low promoter–high gene body” distribution, consistent with reported patterns in Thoroughbred skeletal muscle [24], Arabian horse peripheral blood, and human skeletal muscle [2]. This cross-species conservation suggests that horses maintain the mammalian methylation “framework” under exercise stress, ensuring basal transcriptional accessibility, while subtle differences (e.g., <150 bp CGI boundary shifts) may reflect breed- or tissue-specific selective pressures.

Interestingly, the elite group showed significantly higher global methylation than the ordinary groups, seemingly contradicting the classic observation of PGC-1A promoter demethylation in athlete skeletal muscle [4]. However, our samples were collected from resting blood 24 h before the race, not immediately post-exercise. Human studies have shown that high methylation at rest may reflect “epigenetic memory” of training history—chronic exercise up-regulates DNMT3A, enhancing global maintenance methylation, while key metabolic gene promoters are selectively demethylated by TET enzymes, creating a “global high–local low” mosaic pattern [21]. In our study, 73% of DMRs were in promoters, and elite horses had significantly more hypo-DMRs than hyper-DMRs, consistent with this model: well-trained individuals maintain high background methylation to “lock” non-essential regions while locally demethylating energy and neural pathway genes to keep them primed for activation.

DMR length analysis showed a major peak at 267 bp for CG-DMRs, with 87% ≤500 bp, matching the 250 bp unimodal normal distribution reported in human and mouse blood WGBS, indicating that short DMRs are the basic units of blood methylation variation. Circos plots showed hotspots in the distal regions of chromosomes 1 and 3 (28–30 genes/100 kb), overlapping the speed-related QTL on ECA1 (150–155 Mb) identified in Thoroughbred QTL studies [25], suggesting that blood DMRs may indirectly reflect the genetic background of muscle-related regulatory loci.

Promoter hypomethylation in elite horses was enriched in oxidative phosphorylation, ATP binding, axon guidance and glutamatergic synapse, consistent with post-exercise blood hypomethylation of “mitochondrial organization” GO terms in Arabian horses after 90 km races [26]. Mechanistically, training-induced Ca^2+^-CAMK-PGC-1α signalling can recruit TET2 to PPARGC1A and ND1/ND2 promoters, causing local demethylation [27,28]. We observed concurrent hypomethylation of exons and promoters of ND1/ND2 in blood, which positively correlated with PPARGC1A hypomethylation (r = 0.86), supporting the “long-range co-regulation” hypothesis: mitochondrial and nuclear genes are synchronously activated via common transcription factors (e.g., NRF-1), providing an energy reserve for explosive ATP production during races [29].

Although CHG/CHH-DMRs accounted for only 6–8% of total differences, they were significantly enriched (FDR < 0.01) in axon guidance and protein binding GO terms and were highly coordinated with CG-DMRs (r > 0.86), consistent with the observation in mice that CHH methylation accompanies axonal plasticity [30]. Plant studies have shown that CHH methylation recruits AGO4-siRNA complexes to maintain transposon silencing [31], while recent mammalian studies found that CHH hypomethylation can expose transposon-derived enhancers related to neural development, enabling rapid transcriptional responses. Thus, CHH-DMRs in Yili horse blood may fine-tune neuromuscular coordination genes by “on-off” switching of transposon enhancers to cope with sudden track challenges.

Whether blood DNA methylation can mirror muscle traits has long been debated. However, several studies have shown that exercise-induced methylation signals can be “indirectly recorded” in blood: (i) training muscle releases exosomes carrying methylated fragments into circulation; (ii) muscle metabolites (lactate, β-aminoisobutyric acid) are transported to bone marrow via SLC5A3/SMCT, altering DNMT/TET activity in hematopoietic stem cells and ultimately reflecting in peripheral blood [32]. Blood DMRs identified here for ACTN3, MSTN and FOXO1 were directionally consistent with qMSP results from Thoroughbred skeletal muscle [22] (r = 0.78), suggesting that blood DMRs can serve as non-invasive proxies for performance prediction, although concurrent muscle sampling is still needed to validate causality.

ND1 and ND2 are located in the mitochondrial genome and encode NADH dehydrogenase subunits 1 and 2, respectively—core components of oxidative phosphorylation (OXPHOS) Complex I that drive the first step of the electron transport chain and directly determine ATP synthesis rate and exercise endurance [33]. Human and murine studies have shown that endurance training up-regulates mitochondrial gene expression promoter demethylation, enhancing aerobic capacity. This study identified, in the blood of racehorses, that the coding regions and upstream regulatory regions of the mitochondrial genes ND1/ND2 in the superior-performing group exhibited significant hypomethylation (Δ > 5%, FDR < 0.01). It is important to clarify that ND1/ND2 are genes encoded by mitochondrial DNA (mtDNA), not by the nuclear genome. The conserved regulatory region within the mitochondrial genome—located near the transcriptional start sites of ND1/ND2 on mtDNA—is highly evolutionarily conserved. This hypomethylation pattern suggests that mitochondrial genes enhance transcriptional efficiency through an epigenetic derepression mechanism, thereby increasing oxidative phosphorylation capacity and providing an energetic foundation for the high-intensity exercise performance of Yili horses. Combined with parallel hypomethylation signals at PPARGC1A (master regulator of mitochondrial biogenesis) and NR3C1 (key receptor for stress recovery), these genes form a dual-axis “energy-stress” candidate panel for non-invasive blood-based epigenetic selection or dCas9-TET1-directed demethylation training enhancement [28].

In summary, pre-race blood from Yili horses exhibits a mosaic pattern of “global hypermethylation and focal promoter hypomethylation,” which primes the energy-neural dual-axis pathway (PPARGC1A/ND1/ND2 and axon guidance) and provides an epigenetically pre-activated state for superior athletic performance [34]. These findings not only expand equine exercise epigenetics theory but also deliver a practical target list for developing non-invasive blood biomarkers and epigenome-editing-based training enhancement.

This article also has several limitations. First, the sample size is relatively small (3 horses each in the elite and average groups). Although whole-genome sequencing provides high-resolution epigenetic information, statistical power remains constrained by the sample size. Future studies should expand the sample size to verify the universality and stability of the identified DMRs. Second, the analysis was based solely on a single pre-race blood sample, without dynamic tracking of methylation changes before and after training or competition, which limits our understanding of epigenetic plasticity and the temporal dimension of exercise adaptation. Furthermore, the study focused primarily on DNA methylation and did not integrate other epigenetic modifications such as histone modifications, making it difficult to comprehensively dissect multi-layer epigenetic regulatory networks. Finally, although there is some evidence supporting the association between blood methylation signals and muscle tissue, multi-tissue comparative studies are still needed to confirm the reliability of blood methylation as a surrogate marker for muscle performance [30,33,35,36].

Future research should carry out longitudinal tracking studies to dynamically monitor methylation changes in Yili horses at different time points throughout training cycles and before/after races, revealing the dynamic response patterns of epigenetic markers and their temporal relationship with athletic performance. Secondly, integrating multi-omics data (such as transcriptomics, proteomics, metabolomics) with epigenomic data would help construct a systems-biology model of exercise adaptation in Yili horses and elucidate the functional mechanisms by which methylation regulates gene expression. Moreover, based on the candidate DMRs screened in this study, cost-effective and highly sensitive targeted methylation detection methods (e.g., methylation-specific PCR, targeted sequencing) could be developed to promote their application in practical breeding and training evaluation. Finally, combining gene-editing tools (e.g., dCas9-TET/DNMT systems) or epigenetic drug interventions in cellular or animal models to validate the function of key DMRs would provide experimental evidence for epigenetic-assisted breeding and training optimisation.

## 5. Conclusions

Employing whole-genome bisulfite sequencing (WGBS), we generated for the first time a single-base-resolution methylation profile of pre-race blood samples from Yili horses and systematically compared the epigenetic architecture between elite and ordinary-performing individuals. A total of over 10,000 CG-biassed differentially methylated regions (DMRs) were identified, the majority of which were located in promoter and CpG island regions and were linked to 7221 differentially methylated genes (DMGs). These DMGs were significantly enriched in biological processes crucial for exercise adaptation, including oxidative phosphorylation, protein binding, axon guidance, glutamatergic synapse, and Hedgehog signalling pathway. Although elite horses exhibited higher global methylation levels, the promoters of key metabolic and neuroplasticity-related genes were selectively hypomethylated, suggesting an “epigenetically primed” state. We prioritised six performance-related candidate genes—ACTN3, MSTN, FOXO1, PPARGC1A, ND1, and ND2—which may serve as targets for molecular marker development and epigenetic intervention.

Collectively, our findings demonstrate that variation in athletic performance among Yili horses possesses a quantifiable epigenetic basis; DNA methylation fine-tunes exercise-related traits by modulating genes involved in energy metabolism and neuromuscular plasticity. The promoter-hypomethylated DMR panel established in this study holds potential for early performance prediction and directional selection, laying a solid foundation for deciphering the epigenetic mechanisms underlying equine athletic capacity and advancing molecular marker-assisted breeding programmes.

## Figures and Tables

**Table 1:** Raw Data Quality Control Statistics.

Sample	Raw_Reads	Raw_Bases (G)	Clean_Reads	Clean_Bases (G)	Clean_Ratio (%)	Q20 (%)	Q30 (%)	GC (%)	BS Conversion Rate (%)
O1	426397184	127.92	414833505	114.10	89.20	99.46	96.88	22.68	99.652
O2	343190825	102.96	333107670	91.46	88.83	99.40	96.69	22.71	99.633
O3	437635139	131.29	426609066	117.41	89.43	99.48	96.95	22.45	99.637
E1	303193568	90.96	299025751	82.31	90.49	98.60	96.01	23.18	99.654
E2	302926484	90.88	298969472	82.25	90.50	98.62	96.09	23.31	99.645
E3	300117737	90.04	296339696	81.55	90.57	98.65	96.13	23.13	99.669

## Data Availability

The datasets presented in this study have been deposited in public repositories. The whole-genome methylation sequencing data of horse blood are available in the NCBI BioProject database under accession number PRJNA1250149 (accessible at https://www.ncbi.nlm.nih.gov/, accessed on 8 January 2026). Additional data used and analysed during this study are available from the corresponding author upon reasonable request.

## Supplementary Materials

The following supporting information can be downloaded at: https://www.mdpi.com/article/10.3390/ani16020302/s1, Note that due to network issues, the link may not be accessible at the moment. If you encounter any problems, please check the validity of the URL and try again later. Table S1: Summary of Extreme Values of Methylation Levels and Densities Across Bins in Different Sequence Contexts; Table S2: Table List of DMR Results: The analysis of DMRs using DSS (DSS-single) yielded the following; Figure S1: Circos visualisation of chromosome-wide methylation density in peripheral-blood DNA from Yili horses O2; Figure S2: Circos visualisation of chromosome-wide methylation density in peripheral-blood DNA from Yili horses O3; Figure S3: Circos visualisation of chromosome-wide methylation density in peripheral-blood DNA from Yili horses E2; Figure S4: Circos visualisation of chromosome-wide methylation density in peripheral-blood DNA from Yili horses E3; Figure S5: Circos representation of chromosome-scale methylation patterns for Yili horse blood samples O2; Figure S6: Circos representation of chromosome-scale methylation patterns for Yili horse blood samples O3; Figure S7: Circos representation of chromosome-scale methylation patterns for Yili horse blood samples E2; Figure S8: Circos representation of chromosome-scale methylation patterns for Yili horse blood samples E3; Figure S9: Distribution of methylation levels within CpG-island (CGI) regions for samples O2; Figure S10: Distribution of methylation levels within CpG-island (CGI) regions for samples O3; Figure S11: Distribution of methylation levels within CpG-island (CGI) regions for samples E2; Figure S12: Distribution of methylation levels within CpG-island (CGI) regions for samples E3; Figure S13: Distribution of methylation levels across distinct genomic features in O2; Figure S14: Distribution of methylation levels across distinct genomic features in O3; Figure S15: Distribution of methylation levels across distinct genomic features in E2; Figure S16: Distribution of methylation levels across distinct genomic features in E3.
