# Supplementary material for: Genome-Wide DNA Methylation Analysis of Performance Variation in the 5000-m Speed Race of Yili Horses
Source: Animals (Basel). 2026 Jan 19;16(2):302. doi: 10.3390/ani16020302 (PMC12838019; doi:10.3390/ani16020302)
Supplement: Supplementary file 1 [file animals-16-00302-s001.zip › animals-4054850-supplementary.pdf]

Contents

Table S1: Summary of Extreme Values of Methylation Levels and Densities Across Bins in Different Sequence Contexts 2
Table S2: Table List of DMR Results:The analysis of DMRs using DSS (DSS-single) yielded the following and list of DMR Annotation Results 3
Figure S1：O2 - Circos visualisation of chromosome-wide methylation density in peripheral-blood DNA from Yili horses O2 4
Figure S2：O3-Circos visualisation of chromosome-wide methylation density in peripheral-blood DNA from Yili horses O3 4
Figure S3：E2 - Circos visualisation of chromosome-wide methylation density in peripheral-blood DNA from Yili horses E2 5
Figure S4：E3 - Circos visualisation of chromosome-wide methylation density in peripheral-blood DNA from Yili horses E3 5
Figure S5：O2 - Circos representation of chromosome-scale methylation patterns for Yili horse blood samples O2 6
Figure S6：O3 - Circos representation of chromosome-scale methylation patterns for Yili horse blood samples O3 6
Figure S7：E2 - Circos representation of chromosome-scale methylation patterns for Yili horse blood samples E2 7
Figure S8：E3 - Circos representation of chromosome-scale methylation patterns for Yili horse blood samples E3 7
Figure S9： O2 - Distribution of methylation levels within CpG-island (CGI) regions for samples O2 8
Figure S10：O3 - Distribution of methylation levels within CpG-island (CGI) regions for samples O3 8
Figure S11：E2 - Distribution of methylation levels within CpG-island (CGI) regions for samples E2 9
Figure S12：E3 - Distribution of methylation levels within CpG-island (CGI) regions for samples E3 9
Figure S13：O2 - Distribution of methylation levels across distinct genomic features in O2 10
Figure S14：O3 - Distribution of methylation levels across distinct genomic features in O3 10
Figure S15：E2 - Distribution of methylation levels across distinct genomic features in E2 11
Figure S16：E3 - Distribution of methylation levels across distinct genomic features in E3 11

Samples	CG_level_max	CG_level_min	CG_density_max	CG_density_min
O1	89. 39	55. 67	99. 26	67. 6
O2	88. 3	55. 75	99. 19	66. 9
O3	87. 87	55. 28	99. 32	66. 24
E1	89. 42	54. 52	98. 97	67. 17
E2	88. 33	54. 06	98. 64	67. 54
E3	87. 56	53. 74	98. 89	67. 11
**Samples**	**CHG_level_max**	**CHG_level_min**	**CHG_density_max**	**CHG_density_min**
O1	0. 57	0. 34	0. 43	0. 07
O2	0. 67	0. 36	0. 4	0. 04
O3	0. 61	0. 37	0. 4	0. 07
E1	0. 55	0. 33	0. 35	0. 05
E2	0. 55	0. 32	0. 41	0. 09
E3	0. 54	0. 32	0. 4	0. 12
**Samples**	**CHH_level_max**	**CHH_level_min**	**CHH_density_max**	**CHH_density_min**
O1	0. 69	0. 34	0. 41	0. 08
O2	0. 62	0. 37	0. 31	0. 03
O3	0. 62	0. 37	0. 38	0. 06
E1	0. 56	0. 33	0. 34	0. 04
E2	0. 57	0. 34	0. 38	0. 08
E3	0. 55	0. 32	0. 42	0. 15

Table S1: Summary of Extreme Values of Methylation Levels and Densities Across Bins in Different Sequence Contexts

(1) Samples: Sample names

(2) CG_level_max: Maximum methylation level of mC sites in CG context within each bin

(3) CG_level_min: Minimum methylation level of mC sites in CG context within each bin

(4) CG_density_max: Maximum methylation density of mC sites in CG context within each bin

(5) CG_density_min: Minimum methylation density of mC sites in CG context within each bin

(6) CHG_level_max: Maximum methylation level of mC sites in CHG context within each bin

(7) CHG_level_min: Minimum methylation level of mC sites in CHG context within each bin

(8) CHG_density_max: Maximum methylation density of mC sites in CHG context within each bin

(9) CHG_density_min: Minimum methylation density of mC sites in CHG context within each bin

(10) CHH_level_max: Maximum methylation level of mC sites in CHH context within each bin

(11) CHH_level_min: Minimum methylation level of mC sites in CHH context within each bin

(12) CHH_density_max: Maximum methylation density of mC sites in CHH context within each bin

(13) CHH_density_min: Minimum methylation density of mC sites in CHH context within each bin

chr	start	end	DMR_length	C_number	C_context
NC_001640.1	2954	3104	151	57	CHH
NC_001640.1	4534	4944	411	135	CHH
NC_001640.1	8335	8719	385	135	CHH
NC_001640.1	8539	8662	124	11	CG

chr	group1_meanMethy	group2_meanMethy	diff.Methy	areaStat	C_context
NC_001640.1	0.0694196846693531	0.0338234961667277	0.0355961885026254	276.63539446601	CHH
NC_001640.1	0.0968908525513756	0.040039540652821	0.0568513118985546	840.010082708898	CHH
NC_001640.1	0.0685625111503786	0.0424262625605979	0.0261362485897808	230.421213364169	CHH
NC_001640.1	0.112163413932969	0.0442607601473258	0.067902653785643	45.5245702196246	CG

chr	start	end	DMR_length	group1_meanMethy	group2_meanMethy
NC_001640.1	2954	3104	151	0.0694196846693531	0.0338234961667277
NC_001640.1	2954	3104	151	0.0694196846693531	0.0338234961667277
NC_001640.1	2954	3104	151	0.0694196846693531	0.0338234961667277
NC_001640.1	2954	3104	151	0.0694196846693531	0.0338234961667277
NC_001640.1	2954	3104	151	0.0694196846693531	0.0338234961667277

chr	diff.Methy	areaStat	C_context	Gene name	region
NC_001640.1	0.0355961885026254	276.63539446601	CHH	rna-NC_001640.1_3727..3795	promoter
NC_001640.1	0.0355961885026254	276.63539446601	CHH	rna-NC_001640.1_3868..3936	promoter
NC_001640.1	0.0355961885026254	276.63539446601	CHH	ND1	promoter
NC_001640.1	0.0355961885026254	276.63539446601	CHH	ND2	exon
NC_001640.1	0.0355961885026254	276.63539446601	CHH	rna-NC_001640.1_4976..5045	promoter

Table S2: Table List of DMR Results:The analysis of DMRs using DSS (DSS-single) yielded the following and list of DMR Annotation Results

chr: Chromosome ID

(2) start: DMR start position

(3) end: DMR end position

(4) DMR_length: Length of DMR

(5) C_number: Number of cytosines in the DMR region

(6) group1_meanMethy: Mean methylation level in the case group

(7) group2_meanMethy: Mean methylation level in the control group

(8) diff.Methy: Differential methylation level

(9) areaStat: Statistical significance value, where a higher absolute value indicates greater regional difference

(10) C_context: Sequence context type

(11) region: Region type

(12) Gene name: For regions associated with a gene ID, this field includes the gene name and relevant description (if available)

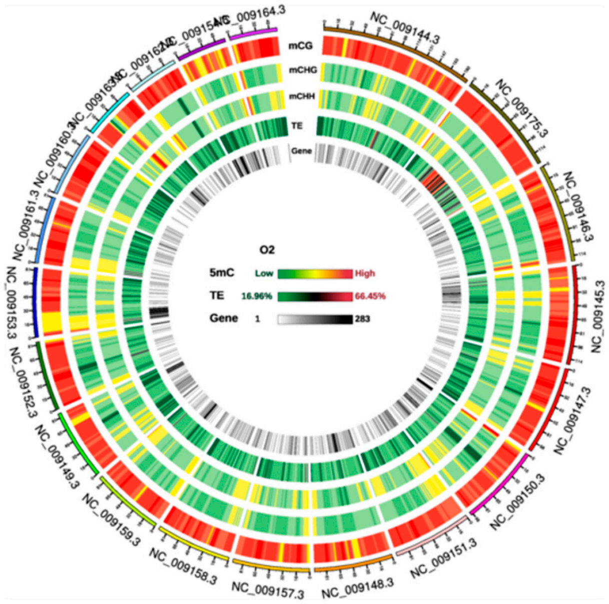

Figure S1： O2-Circos visualisation of chromosome-wide methylation density in peripheral-blood DNA from Yili horses O2

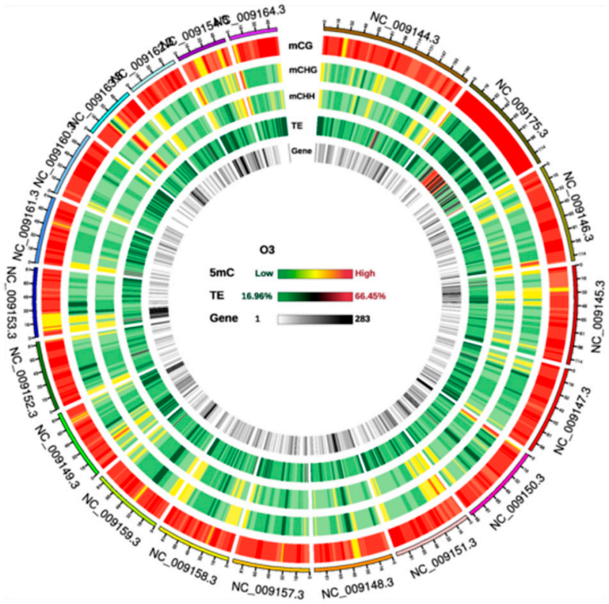

Figure S2：O3-Circos visualisation of chromosome-wide methylation density in peripheral-blood DNA from Yili horses O3

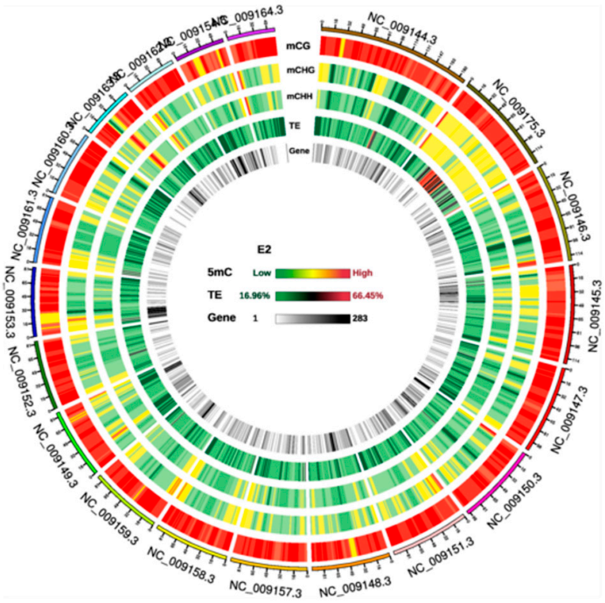

Figure S3：E2-Circos visualisation of chromosome-wide methylation density in peripheral-blood DNA from Yili horses E2

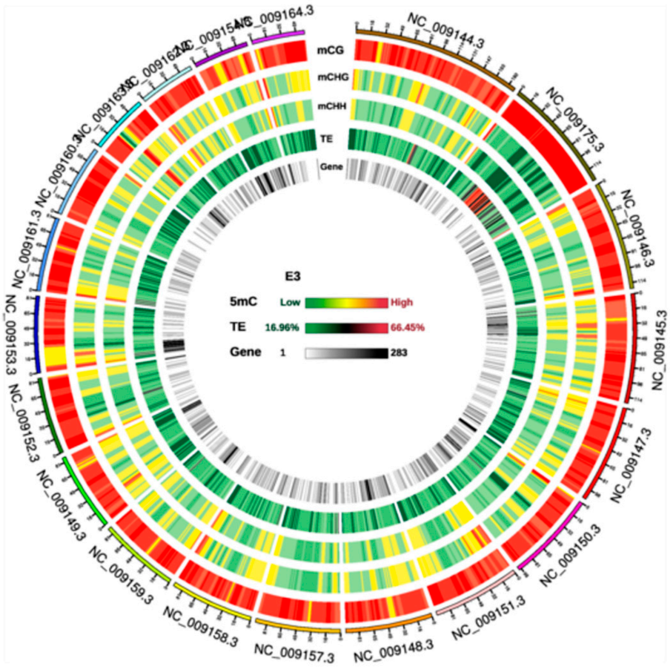

Figure S4：E3-Circos visualisation of chromosome-wide methylation density in peripheral-blood DNA from Yili horses E3

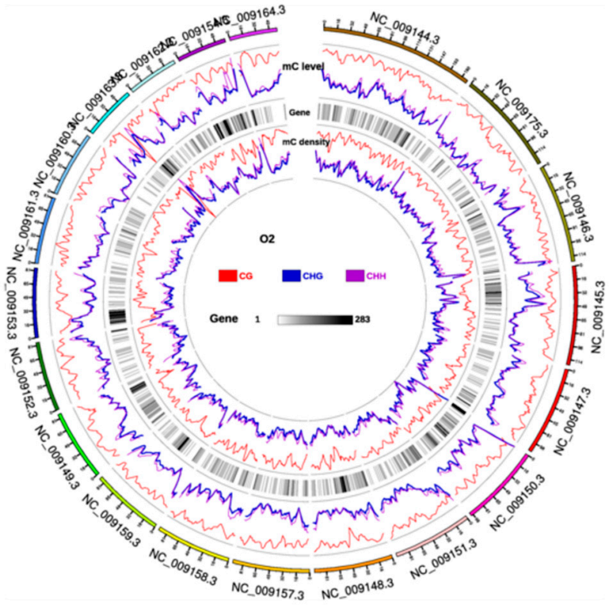

Figure S5：O2-Circos representation of chromosome-scale methylation patterns for Yili horse blood samples O2

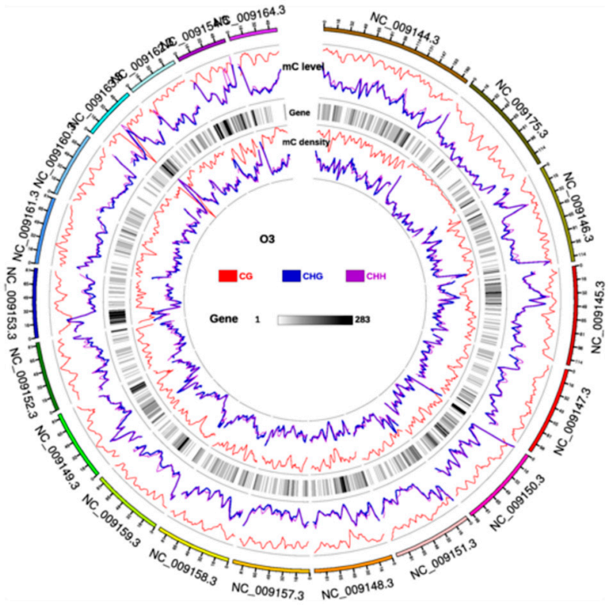

Figure S6：O3-Circos representation of chromosome-scale methylation patterns for Yili horse blood samples O3

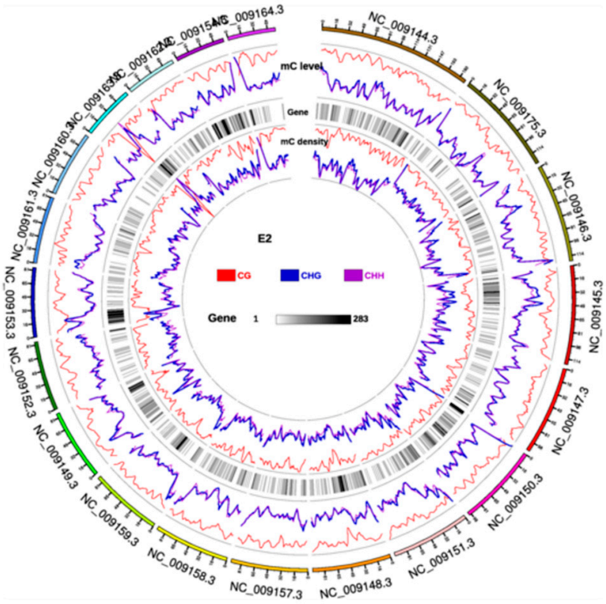

Figure S7：E2-Circos representation of chromosome-scale methylation patterns for Yili horse blood samples E2

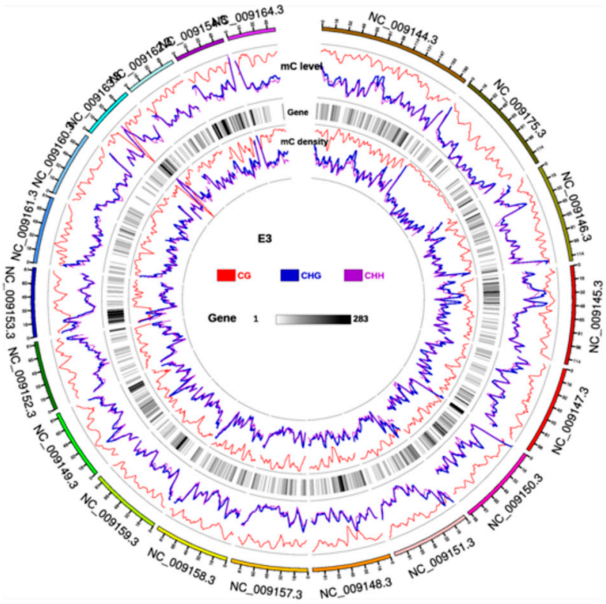

Figure S8：E3-Circos representation of chromosome-scale methylation patterns for Yili horse blood samples E3

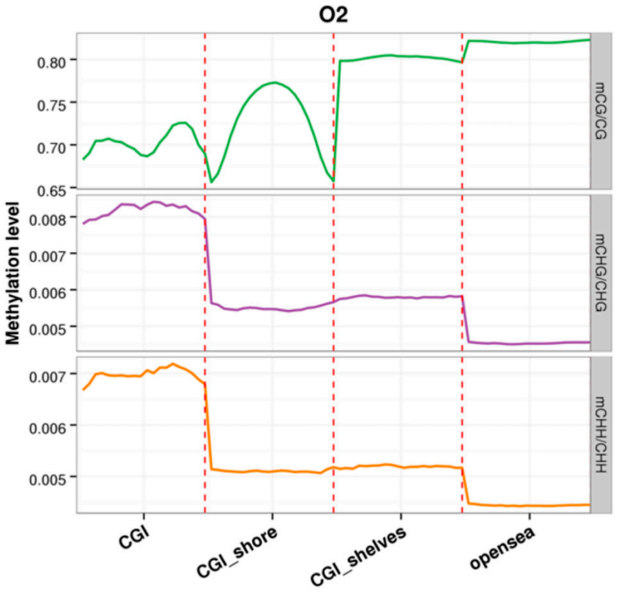

Figure S9：O2-Distribution of methylation levels within CpG-island (CGI) regions for samples O2

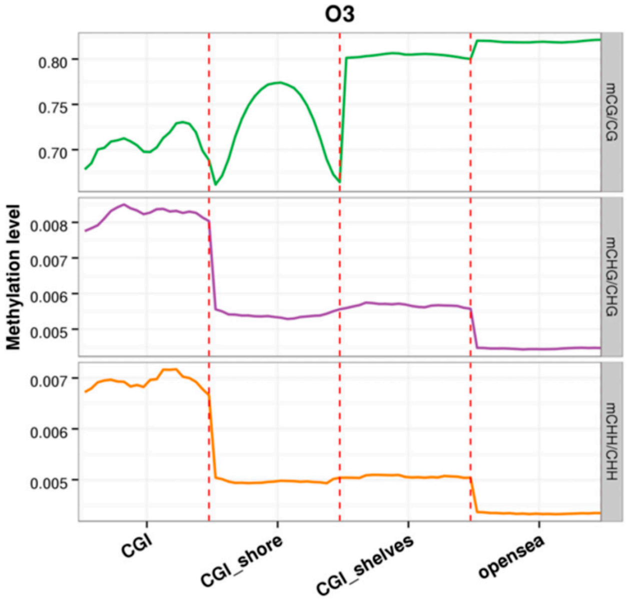

Figure S10： O3-Distribution of methylation levels within CpG-island (CGI) regions for samples O3

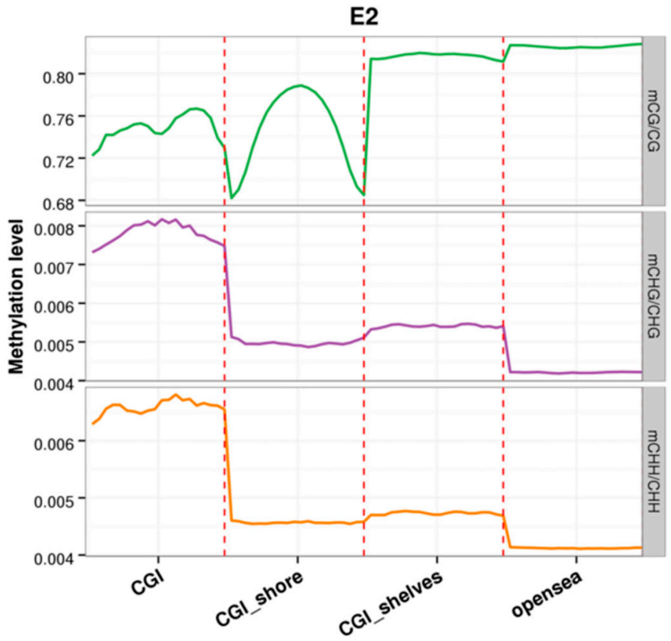

Figure S11：E2-Distribution of methylation levels within CpG-island (CGI) regions for samples E2

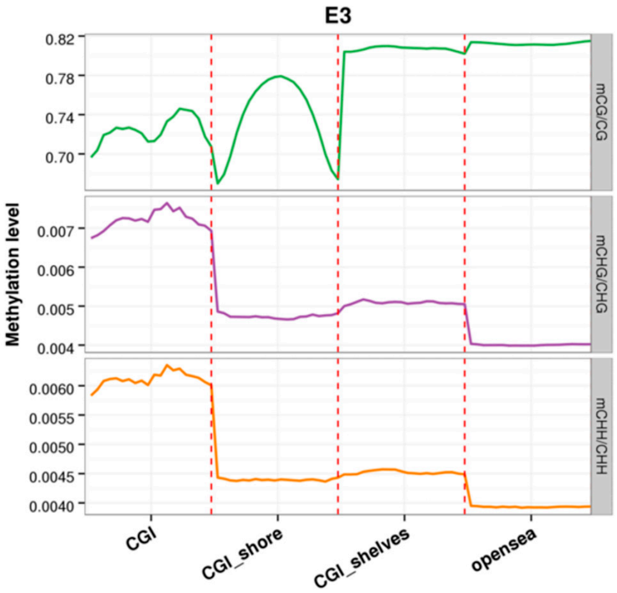

Figure S12：E3-Distribution of methylation levels within CpG-island (CGI) regions for samples E3

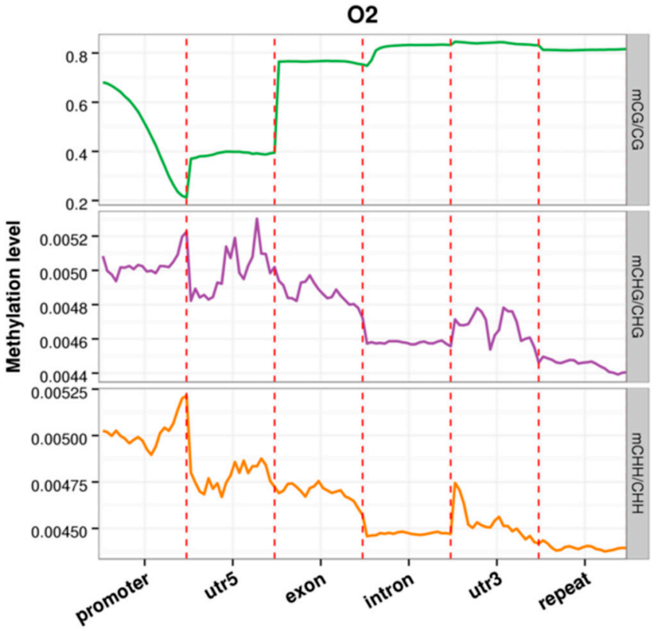

Figure S13：O2-Distribution of methylation levels across distinct genomic features in O2

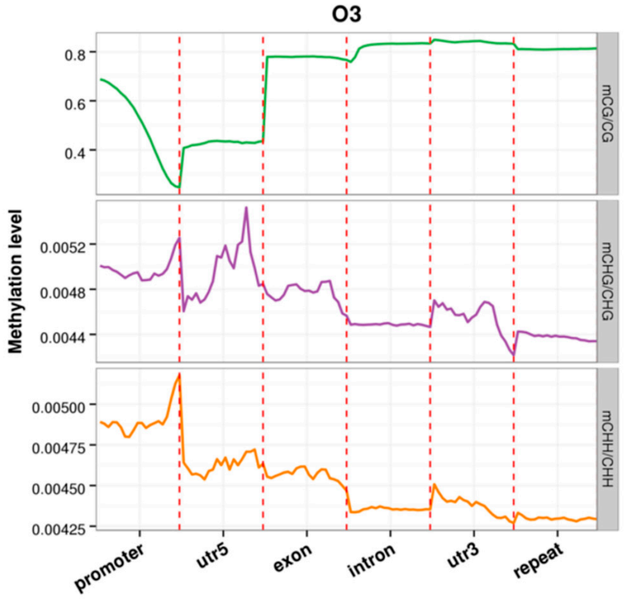

Figure S14：O3-Distribution of methylation levels across distinct genomic features in O3

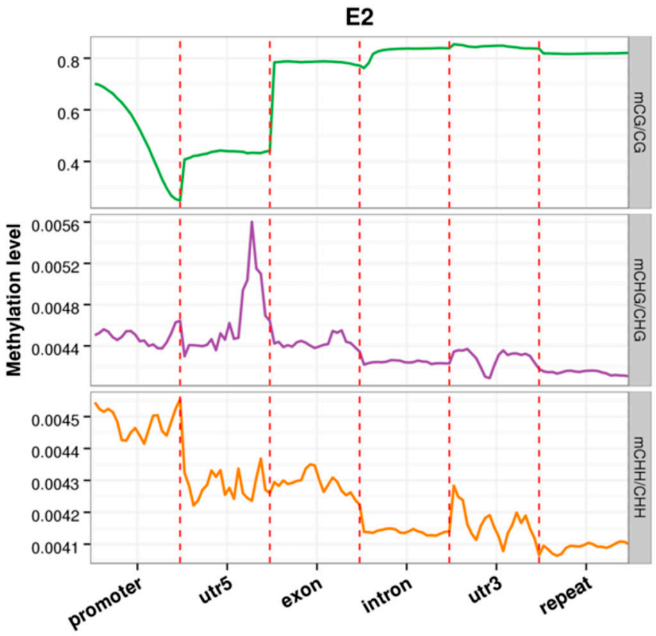

Figure S15： E2-Distribution of methylation levels across distinct genomic features in E2

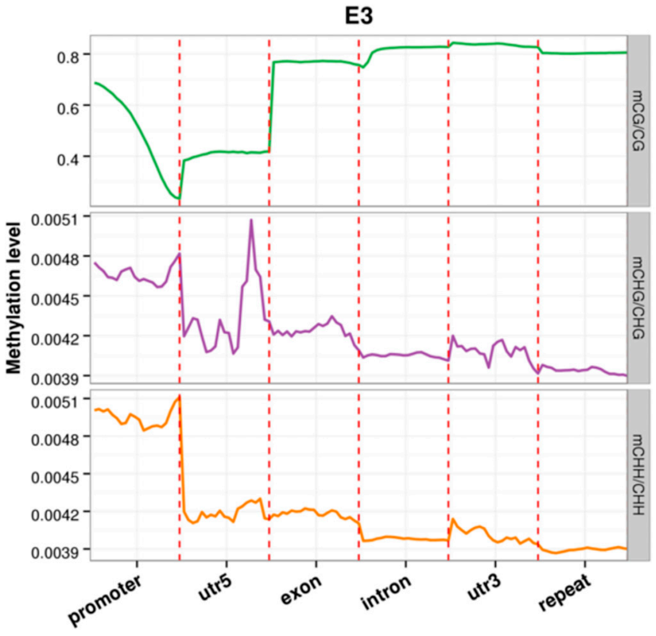

Figure S16： E3-Distribution of methylation levels across distinct genomic features in E3
